# Basicity–Controlled C–H Bond Activation by a Structurally Characterized Ni(III)–Hydroxo Complex

**DOI:** 10.1021/jacs.5c06941

**Published:** 2025-07-25

**Authors:** Hung-Ruei Pan, John Wu, Chun-Ming Tsai, Pei-Juan Liao, Hua-Fen Hsu

**Affiliations:** Department of Chemistry, 34912 National Cheng Kung University, Tainan 701, Taiwan

## Abstract

The selective oxidation of strong C–H bonds remains a central challenge in synthetic chemistry, in part due to the elusive nature of active oxidants and their underlying mechanisms. Herein, we report the isolation and complete characterization of a room–temperature–stable mononuclear Ni­(III)–hydroxo complex, [Na­(15c5)]­[Ni­(PS3″)­(OH)] ([Na­(15c5)]­[**2**]), supported by a *tris*(benzenethiolato)­phosphine ligand derivative. The X-ray crystallographic structure of **2** reveals a trigonal bipyramidal Ni­(III) center, in which the coordinated hydroxo ligand is stabilized by secondary coordination sphere interactions. Complex **2** displays hydrogen atom transfer (HAT) reactivity toward strong C–H bonds, including that in cyclohexane (BDE = 99.5 kcal mol^–1^). Kinetic studies with various C–H substrates reveal a strong linear correlation between log­(*k*_2_) and substrate p*K*_a_, but a poor correlation with C–H bond dissociation energies, indicating an asynchronous PCET pathway with a transition state predominantly governed by proton transfer (PT). The O–H bond dissociation free energy of a resulting Ni­(II)–aqua species was estimated to be 96.6–100.3 kcal mol^–1^ based on thermodynamic data. A semiempirical free energy analysis following the approach of Barman et al. (*Proc. Natl. Acad. Sci. U.S.A.* **2021**, 118, e2108648118) gives a best-fit *x* value of 0.18 (*R* ^2^ = 0.99), where *x* = 1 indicates synchronous PCET and lower values reflect greater PT character in the transition state. These findings underscore the critical role of basicity in modulating PCET reactivity and establish complex **2** as a rare, well–defined Ni­(III)–OH oxidant capable of strong C–H bond activation at low redox potential.

## Graphical abstract

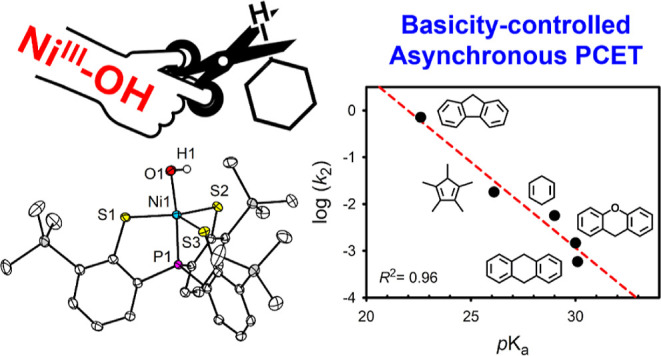

## Introduction

The selective oxidation of strong C–H bonds remains a fundamental challenge in synthetic chemistry, with broad implications for biological transformations and industrial processes.^1–3^ Nickel­(II) complexes have been extensively explored for alkane hydroxylation and olefin epoxidation using oxidants such as *m*–chloroperbenzoic acid (*m*–CPBA), hypochlorite, and hydrogen peroxide.^4–12^ Despite these advances, the identities of the active oxidants and the mechanistic pathways involved in Ni–catalyzed C–H activation remain poorly defined. Proposed key intermediates include Ni­(II)–O^•‑^,^13^ Ni­(III)–O^•‑^,^14^ Ni­(IV)=O, and Ni­(III)–OH species,^15,16^ although some studies suggest alternative radical–based pathways that do not involve direct C–H activation by terminal Ni–O species.^17^ To gain mechanistic insight into these reactions, several Ni–oxygen species, including Ni­(III)–O^•^ (Scheme 1A,B),^18,19^ Ni­(III)–O­(H) (Scheme 1C),^16^ and Ni­(III)–O···H···OAc (Scheme 1D),^20^ have been generated in situ and characterized spectroscopically. Dinuclear Ni­(III) and Ni­(IV) complexes, such as bis−μ–oxo and tris−μ–oxo species, also exhibit oxidative reactivity.^21,22^ High–valent Ni–X complexes (X = OAc,^23^ OCl,^10,24^ ONO_2_,^23,25^ Cl,^26–28^ and HCO_3_)^23,29^ further underscore the potential of Ni–based oxidants. However, isolable and well–characterized mononuclear Ni­(III)–OH complexes capable of C–H activation remain exceedingly rare.

**Scheme 1:**
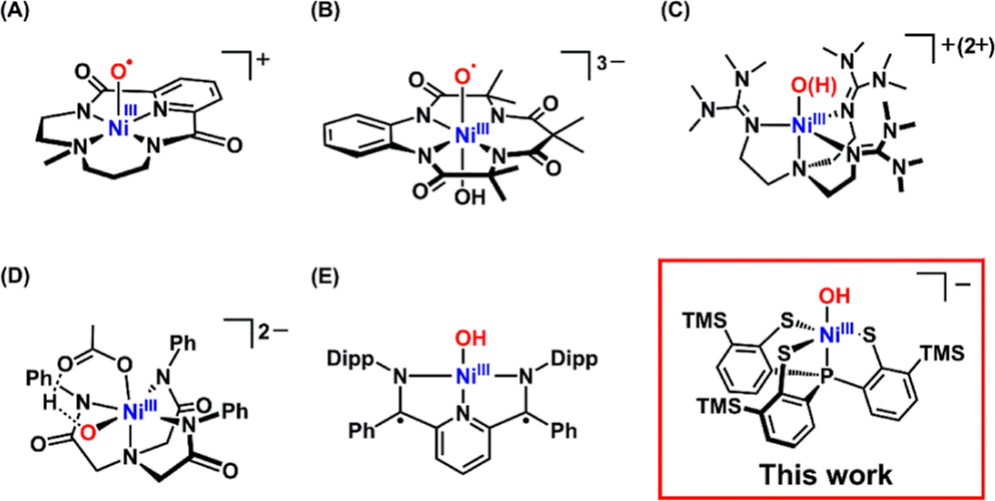
Representative High–Valent Ni–Oxygen Complexes Exhibiting HAT Reactivity

Herein, we report the isolation and X–ray characterization of a room–temperature–stable mononuclear Ni­(III)–hydroxo complex, [Na­(15c5)]­[Ni­(PS3″)­(OH)] ([Na­(15c5)]­[**2**]), supported by a *tris*(benzenethiolato)­phosphine ligand derivative. To date, only two structurally characterized Ni­(III)–OH complexes have been reported, but their reactivity has not been established,^25^ or remains insufficiently explored (Scheme 1E).^30^ In contrast, complex **2** exhibits hydrogen atom transfer (HAT) reactivity toward strong C–H bonds. Such reactivity is often mediated by high–valent metal–oxo or −hydroxo species and is increasingly understood through the lens of proton–coupled electron transfer (PCET), particularly its asynchronous variant wherein proton transfer (PT) and electron transfer (ET) occur in a concerted but thermodynamically unbalanced fashion.^31,32^ Recent works have shown that the basicity of metal–oxo or −hydroxo units can strongly influence the kinetics and thermodynamics of C–H bond cleavage via asynchronous PCET, as demonstrated for Mn­(IV)–oxo,^33^ Co­(III)–oxo,^34,35^ and Mn­(III)–hydroxo complexes.^36^ In contrast, Ru­(IV)–oxo and Fe­(III)–hydroxo species display oxidative asynchronous PCET pathways.^37,38^ Building on these insights, we demonstrate that the high basicity of the Ni­(III)–OH unit in complex **2** governs its reactivity, enabling activation of strong C–H bonds through a basicity–controlled asynchronous PCET mechanism.

## Results and Discussion

### Synthesis and Characterization

A penta–coordinate Ni­(III)–DABCO complex, [Ni­(PS3″)­(DABCO)] (**1**), was synthesized by reacting the previously reported dinuclear Ni­(III) complex, [Ni^III^(PS3″)_2_],^39^ with excess DABCO in THF. After solvent removal, the residue was redissolved in CH_2_Cl_2_ and layered with CH_3_OH, affording block–like crystals of **1**·CH_2_Cl_2_ in 72% yield. Complex **1** was further reacted with NaOH and 15–crown–5 (15c5) in THF, yielding a dark green solution. Layering with hexane produced plate–like crystals of [Na­(15c5)]­[Ni­(PS3″)­(OH)]·2THF·H_2_O ([Na­(15c5)]­[**2**]·2THF·H_2_O) in 63% yield (Scheme 2).

**Scheme 2:**
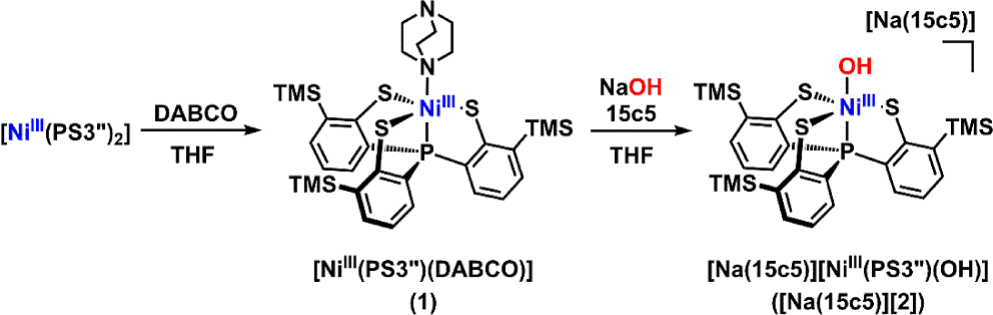
Synthetic Route to Complex **1** and [Na­(15c5)]­[**2**]

The molecular structures of complexes **1** and **2** were determined by single–crystal X–ray diffraction (Figure 1; Tables S1–S4). Complex **1** adopts a slightly distorted trigonal bipyramidal geometry around the nickel center (τ_5_ = 0.88), whereas complex **2** exhibits a τ_5_ value of 0.54, indicative of a geometry intermediate between trigonal bipyramidal and square pyramidal.^40^ Both complexes display comparable average Ni–S bond lengths, with Ni–S_avg_ values of 2.25 Å and 2.28 Å, respectively. The Ni–OH bond length in **2** is 1.892(3) Å, slightly longer than those reported for other Ni­(III)–OH species (1.824 Å and 1.829 Å),^25,30^ possibly due to interaction with the sodium cation. The hydroxo H–atom was located in the Fourier difference map, and short O1···S distances (3.04–3.23 Å) suggest intramolecular hydrogen bonding with the sulfur donors. Infrared (IR) spectroscopy further supports the presence of the hydroxo group, with an O–H stretching band at 3606 cm^–1^ that shifts to 2727 cm^–1^ upon deuterium substitution (Figure S1). Ni K–edge X–ray absorption spectroscopy (XAS) of **1** and **2** shows nearly identical pre–edge features at 8333.6 and 8333.4 eV, respectively (Figure S2), consistent with similar electronic structures for the Ni­(III) centers in both complexes.

**Figure 1:**
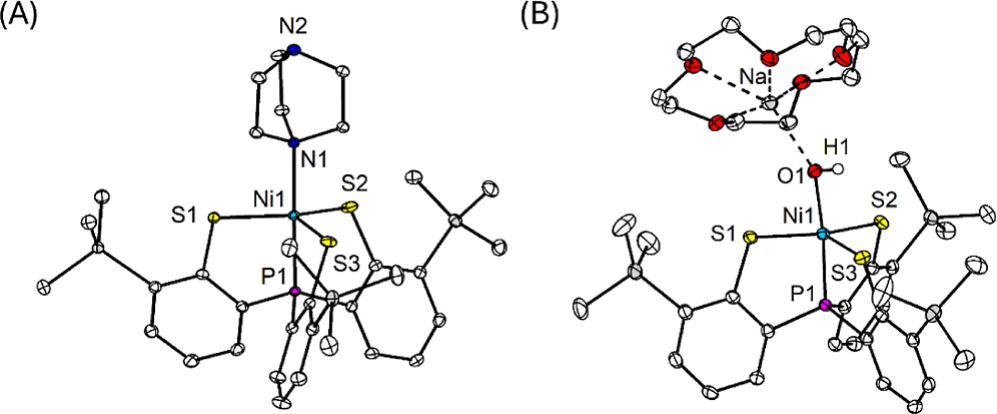
ORTEP diagrams of (A) **1**·CH_2_Cl_2_ and (B) [Na­(15c5)]­[**2**]·2THF·H_2_O with thermal ellipsoids shown at 35% probability. Solvent molecules and hydrogen atoms are omitted for clarity, except for the H–atom (H1) in the hydroxo group.

The electronic absorption spectrum of complex **1** displays three characteristic bands at 615, 734, and 1023 nm (Figure S3), while complex **2** exhibits two prominent bands at 585 and 733 nm, along with a broad plateau in the near–IR region (Figure 2A). The ^1^H nuclear magnetic resonance (NMR) spectra of both complexes reveal three paramagnetically shifted signals attributed to the phenyl protons of the PS3″ ligands (16.75, 10.33, and −8.25 ppm for **1**; 15.12, 8.95, and −4.01 ppm for **2**), consistent with a *C*_3_–symmetric environment (Figures S4 and S5). This symmetry is further supported by the presence of a single resonance for the trimethylsilyl groups, observed at 1.91 ppm for **1** and 2.55 ppm for **2**. The paramagnetic properties of complexes **1** and **2** were investigated using the Evans method, yielding effective magnetic moments (μ_eff_) of 1.68 and 1.62 μ_B_, respectively, indicating an *S* = 1/2 spin state. Correspondingly, X–band electron paramagnetic resonance (EPR) spectra of **1** and **2** in 2–MeTHF at 77K display rhombic signals centered around *g* ≈ 2. Simulations of the spectra gave *g* values of 2.40, 2.04, and 2.01 for **1**, and 2.33, 2.09, and 2.04 for **2** (Figures S6 and S7). These data are consistent with low–spin d^7^ Ni­(III) centers in both complexes. Furthermore, Rotating–frame Overhauser Effect Spectroscopy (ROESY) was performed to probe the interaction between the bound hydroxide and [Na­(15c5)]^+^ in solution.^41^ The absence of cross–peaks between the trimethylsilyl group and the crown ether moiety indicates a lack of through–space coupling (Figure S8). These results suggest that the weak interaction observed in the solid–state structure does not persist in solution, likely due to dynamic solvation or ion–pair dissociation.

**Figure 2:**
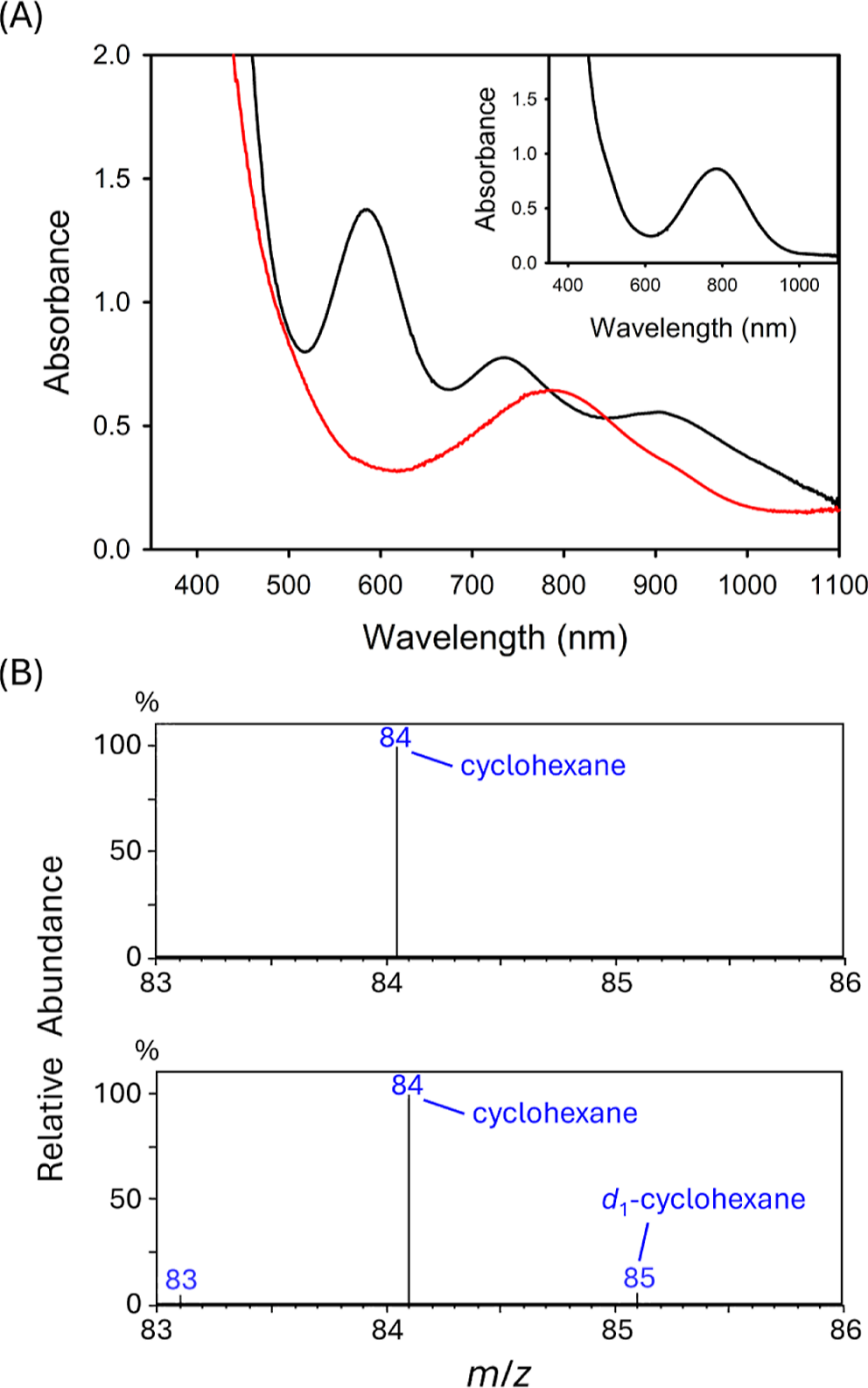
(A) UV–vis–NIR spectra of [Na­(15c5)]­[**2**] (0.6 mM) before (black) and after reaction with 200 equiv of cyclohexane (red) in DMSO. Inset: spectrum of [Na­(15c5)]­[**2**] after treatment with 1 equiv. each of [2,6–LutH]­[BAr^F^_4_] and CoCp*_2_. (B) GC–MS spectra of post–reaction mixtures of [Na­(15c5)]­[**2**] (0.015 M) and 200 equiv of cyclohexane in DMSO (top) and *d*_6_–DMSO (bottom) after 5 days at room temperature.

### Reactivity Studies

As described earlier, high–valent Ni–oxygen species such as Ni­(III)–O^•–^, Ni­(IV)=O, and Ni­(III)–OH have been proposed as key intermediates in the hydroxylation of aliphatic C–H bonds catalyzed by Ni­(II) complexes in the presence of oxidants such as *m*–CPBA acid or hypochlorite. However, direct evidence for these species remains elusive, as they have not been isolated and lack definitive structural characterization. To address this, we employed complex **2**, a structurally characterized Ni­(III)–OH species, to evaluate its HAT reactivity toward strong C–H bond substrates such as cyclohexane (bond dissociation energy (BDE) = 99.5 kcal mol^–1^) and toluene (BDE = 90.9 kcal mol^–1^).^42^ Complex **2** remains stable in DMSO at room temperature for several days. However, treatment with cyclohexane gradually turns the dark green solution yellowish brown over the course of 3–5 days. The ultraviolet–visible–near-infrared (UV–vis–NIR) spectrum of the resulting mixture displays a broad absorption band centered at 780 nm (Figure 2). The ^1^H NMR spectrum exhibits resonances consistent with the formation of [Ni^II^(PS3″)­(H_2_O)]^−^ (**3**), including a singlet at 0.35 ppm assigned to the Si­(CH_3_)_3_ groups and three well–resolved aromatic signals at 6.76, 7.14, and 7.21 ppm, attributable to the PS3″ ligand (Figure S9). The ^31^P NMR spectrum shows a sharp resonance at 83.6 ppm (Figure S10). The combined ^1^H and ^31^P NMR data confirm the diamagnetic nature of complex **3** and support a *C*_3_–symmetric coordination environment at the nickel­(II) center. Similar spectroscopic features were observed upon independent treatment of **2** with 1 equiv of CoCp*_2_ and [2,6–LutH]­[BAr^F^_4_] (Figure 2A inset; Figures S11 and S12), further supporting the formation of a corresponding Ni­(II)–OH_2_ species in the reaction of **2** with cyclohexane via a HAT mechanism. The resulting cyclohexyl radical is likely quenched by DMSO, as evidenced by the detection of the [C_6_H_12_]+1 ion (*m*/*z* = 85) in the gas chromatography–mass spectrometry (GC–MS) spectrum when the reaction is performed in *d*_6_–DMSO (Figure 2B). Notably, this peak is absent under identical conditions in non–deuterated DMSO, consistent with incorporation of a deuterium atom during radical quenching. Comparable results were obtained with toluene as the substrate, as demonstrated by spectroscopic analysis (Figures S13 and S14).

Although the PCET reactivity of complex **2** with cyclohexane and toluene was observed, the sluggish reaction rates under experimental conditions prevented accurate determination of kinetic parameters. To further evaluate the C–H bond activation reactivity of complex **2**, a series of substrates with relatively weaker C–H bonds was examined in DMSO at 35 °C to establish their kinetic profiles (Table 1). Upon addition of substrates, the dark–green solution of **2** gradually turns yellowish–brown, and the resulting UV–vis–NIR and ^1^H NMR spectra closely matched those observed upon treatment of **2** with 1 equiv. each of CoCp*_2_ and [2,6–LutH]­[BAr^F^_4_], consistent with the formation of the corresponding Ni­(II)–OH_2_, complex **3** (Figures 3 and S15–S19). In all cases, the reactions proceeded nearly quantitatively, and the organic products were identified by ^1^H NMR spectroscopy (Figures S15–S19; Tables S5 and S6).

**Table 1:** Thermodynamic Parameters and Kinetic Data for 2 and Various Substrates of C–H Activation

Substrate	BDE^a^	*p*K_a_	*k*_2_ ^b^
indene	83.0	20.1	n.d^c^
fluorene	82.0	22.6	8.70 × 10^–1^
HCp*	77.0	26.1	1.92 × 10^–2^
1,4–CHD	76.0	∼29^d^	7.87 × 10^–3^
xanthene	75.2	30.0	1.26 × 10^–3^
9,10–DHA	76.3	30.1	5.02 × 10^–4^

a BDE in kcal mol^–1^.

b *k*_2_ in M^–1^ s^–1^.

c n.d. indicates not detected.

d The p*K*_a_ value of 1,4–CHD is not available in the literature. It was estimated based on the linear correlation between *k*_2_ and p*K*_a_ values for various substrates, as reported in the study by Barman et al.^33^

**Figure 3:**
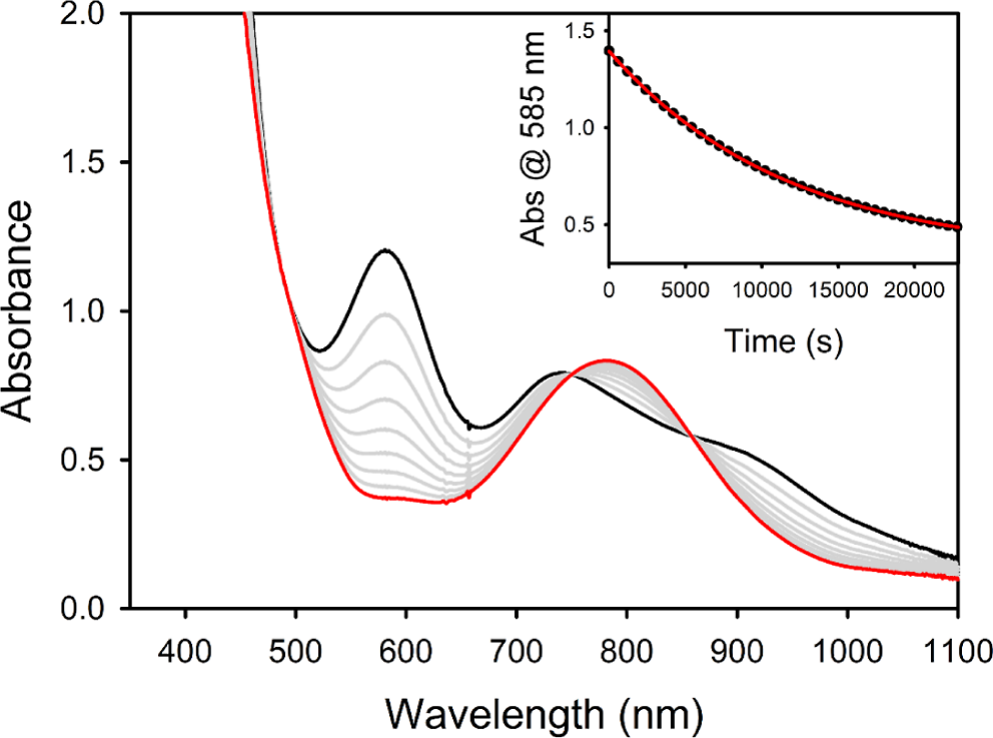
UV–vis–NIR spectral change of [Na­(15c5)]­[**2**] (0.6 mM) with 9,10–DHA (0.0561 M) in DMSO at 35 °C. Initial: black line; final: red line. Inset: time–dependent change of absorbance at 585 nm. Black dot: experimental data; red line: nonlinear least-squares fit for exponential decay.

As a representative example, the reaction with 9,10–DHA exhibited a clean spectral transformation with well–defined isosbestic points at 750 and 858 nm, indicating a direct conversion without detectable intermediates (Scheme 3 and Figure 3). Monitoring the decay of the 585 nm band revealed exponential behavior, consistent with a pseudo–first–order process. A second–order rate constant (*k*_2_) of 5.02 × 10^–4^ M^–1^ s^–1^ was determined from the linear relationship between *k*_obs_ and [9,10–DHA], suggesting that complex **2** possesses modest oxidizing power (Figure S15 and Table S5). The oxidized products, anthracene and 9,9′,10,10’–tetrahydro–9,9’–bianthryl, were identified by ^1^H NMR spectroscopy, with yields of 63% and 25%, respectively, based on ^1^H NMR peak integration (Figure S15 and Table S6). Additionally, ^31^P NMR spectrum shows a sharp signal at 83.5 ppm, which is in agreement with formation of **3** vide supra (Figure S20). Interestingly, the reaction of **2** with fluorene afforded a second–order rate constant of 8.70 × 10^–1^ M^–1^ s^–1^, 3 orders of magnitude greater than that observed with 9,10–DHA, despite fluorene having a significantly higher BDE (82.0 kcal mol^–1^) compared to 9,10–DHA (BDE = 76.3 kcal mol^–1^).^42^ The reaction with *d*_10_–fluorene yielded a kinetic isotope effect (KIE) of 5.1, consistent with a PCET mechanism in the rate–determining step (Figure S16 and Tables S5 and S6). However, the inverse correlation between *k*_2_ and BDE observed for these two substrates deviates from the expected thermodynamic trend.

**Scheme 3:**
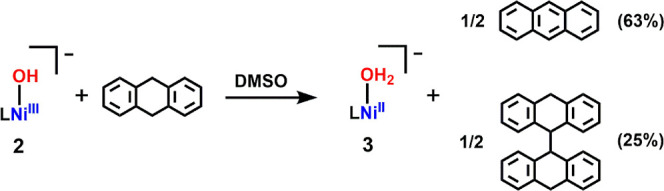
Reaction of Complex **2** With 9,10–DHA in DMSO

This apparent contradiction may stem from an asynchronous PCET mechanism, in which the electron and proton transfers are energetically decoupled. In such cases, factors beyond BDE such as transition–state basicity or redox potential can play dominant roles in governing the rate of C–H bond cleavage. Notably, the observed *k*_2_ values showed poor correlation with substrate BDEs (Figure S21). Instead, a strong linear relationship was found between log (*k*_2_) and substrate p*K*_a_ (*R* ^2^ = 0.94), with *k*_2_ increasing as p*K*_a_ decreases (Figure 4A).^43,44^ These results support an imbalanced transition state and a p*K*_a_–driven asynchronous PCET mechanism. In addition, the reaction with indene proceeded within seconds under the same conditions, attributed to its high acidity (p*K*_a_ = 20.1) despite its relatively strong C–H bond (BDE = 83.0 kcal mol^–1^).^42^ Although the reaction was too fast to determine a reliable *k*_2_ value, the formation of complex **3** as the final product supports a PCET pathway (Figure S22).

**Figure 4:**
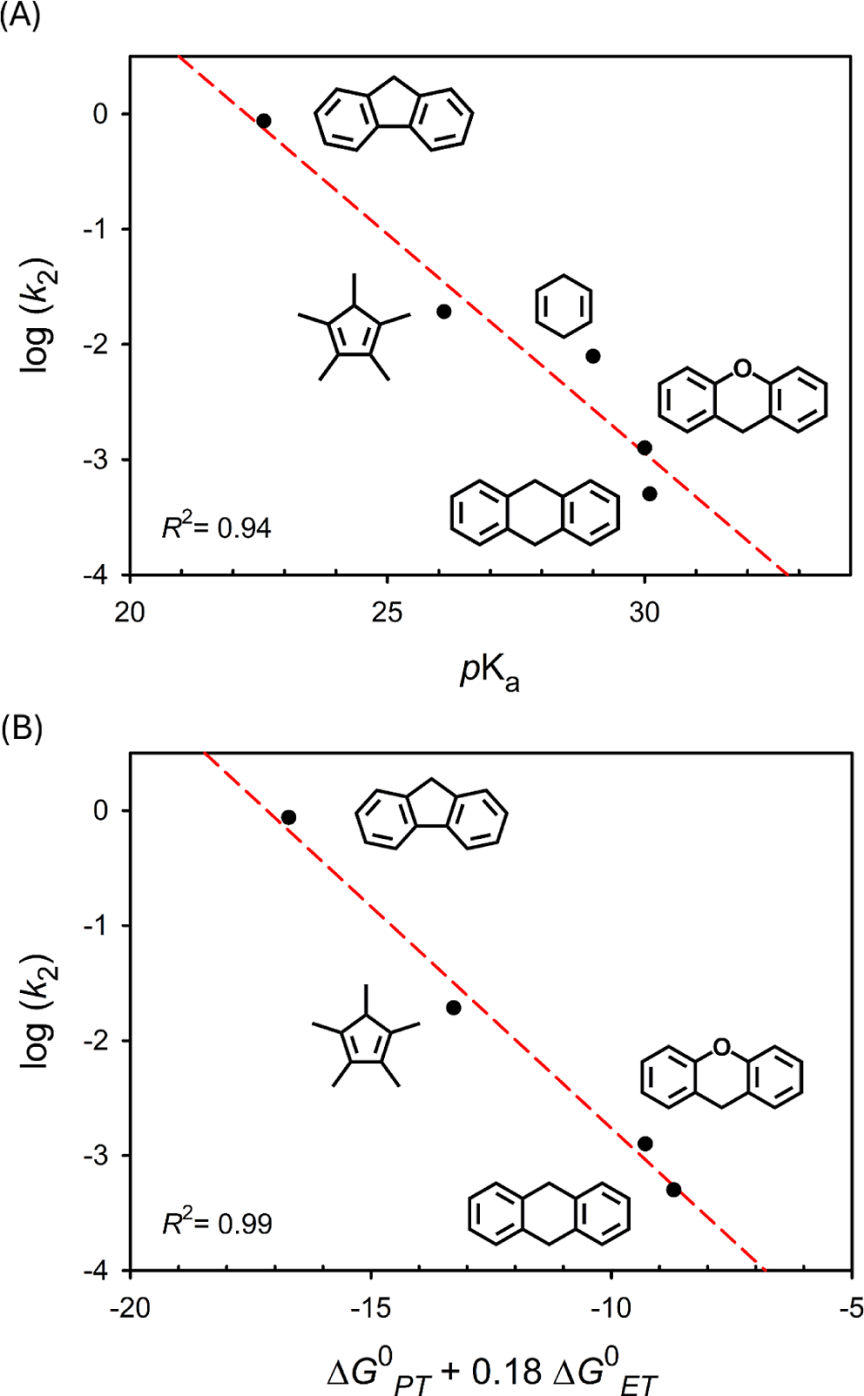
Plots of log (*k*_2_) versus (A) p*K*_a_ and (B) Δ*G* ^0^_PT_ + 0.18 Δ*G* ^0^_ET_.

To assess the thermodynamic driving force, the bond dissociation free energy (BDFE) of the O–H bond in complex **3** was estimated using the Bordwell eq (eq 1, Scheme 4). Treatment of **2** with [2,6–LutH]­[BAr^F^_4_] generated a new species exhibiting distinct absorption bands at 622, 750, and 990 nm in the UV–vis–NIR spectrum (Figure S23). The ^1^H NMR data confirmed the formation of a paramagnetic Ni species, assigned as the protonated complex, **2–H** ^**+**^ (Figure S24). Complex **2** remains stable in DMSO (p*K*_a_ = 35), but upon addition of propionitrile (p*K*_a_ = 32.5),^45^ the UV–vis–NIR spectrum of **2–H** ^**+**^ was reproduced (Figure S25), indicating that the p*K*_a_ of **2–H** ^**+**^ lies between 32.5 and 35. Cyclic voltammetry of in situ generated **2–H** ^**+**^ revealed an irreversible reduction wave at −0.68 V vs Fc/Fc^+^, assigned to the **2–H** ^**+**^/**3** redox couple (Figure S26). Using the experimentally determined p*K*_a_ and *E*_1/2_ values and free energy constant *C*_G_ (68 kcal mol^–1^ in DMSO),^43^ the BDFE of the O–H bond in **3** was estimated to range from 96.6 to 100.3 kcal mol^–1^ (eq 1), consistent with its ability to oxidize strong C–H bonds such toluene and cyclohexane.

1 $$BDFE=1.37{\;pK}_{a}+23.06\;E^{0}+C_{G}$$

**Scheme 4:**
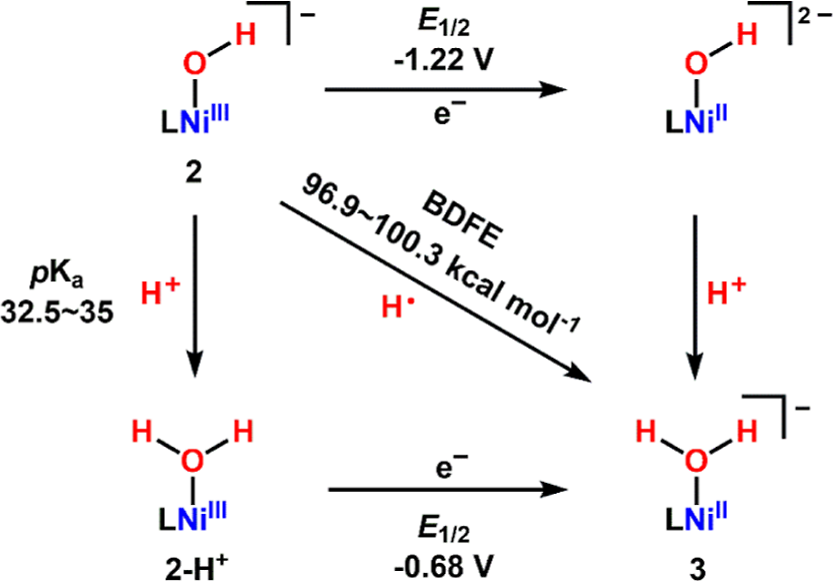
Thermodynamic Square of 2

Notably, the conjugate acid of complex **2** exhibits a remarkably high p*K*_a_ value. Its ability to activate strong C–H bonds is attributed to its high basicity, which effectively compensates for its relatively low redox potential. This balance enables HAT via a PT–dominated, asynchronous PCET mechanism. To assess the relative contributions of proton and electron transfer, we employed the asynchronicity factor (η), as defined by Srnec and co-workers, which is based on the difference in reduction potentials and p*K*_a_ values between the oxidant (Ni­(III)–OH) and the radical conjugate of the substrate (R^•^).^31^ All examined substrates exhibited negative η values, ranging from −1.63 to −2.30,^46^ consistent with a PT–dominated transition state (Table S7; see Supporting Information for details).

To better quantify the impact of basicity and redox potential on reactivity, we applied the semiempirical model developed by Barman et al., which relates the activation free energy (Δ*G* ^‡^) to a weighted sum of Δ*G* ^0^_PT_ and *x*Δ*G* ^0^_ET_ (eq 2 and Table S8, details in SI)^33^

2 $$\log k_{2}=-\left(\frac{\alpha }{2.303RT}\right)\left(\Delta G_{PT}^{0}+x\Delta G_{ET}^{0}\right)+\beta^{\prime }$$

An *x* value of 1 corresponds to a fully synchronous PCET process, whereas values less than 1 indicate increasing PT character in the transition state. Accordingly, two previously reported systems exhibiting basicity–controlled PCET reactivity, [Mn^IV^H_3_buea­(O)]^−^ and PhB­(^*t*^BuIm)­Co^III^O, yield *x* values of 0.56 and 0.45, respectively, based on the approach of Barman et al.^33,34,47^ In this Ni­(III)–OH system, the best fit of the kinetic data afforded an *x* value of 0.18 (*R* ^2^ = 0.99), indicating a transition state highly dominated by proton transfer (Figures 4B and S27). The redox potential of 1,4–CHD in DMSO has not been reported and was therefore excluded from the fitting. Collectively, these findings identify complex **2** as a rare example of a high–valent nickel–hydroxo species that promotes strong C–H bond activation via a basicity–controlled, asynchronous PCET pathway. This mechanistic paradigm highlights how high basicity of metal oxygen species can be strategically leveraged to achieve oxidative reactivity at low redox potential.

## Conclusion

This work reports the isolation and full characterization of a mononuclear Ni­(III)–hydroxo complex, [Na­(15c5)]­[Ni­(PS3″)­(OH)] ([Na­(15c5)]­[**2**]), representing a rare, structurally defined high–valent nickel–oxygen species capable of activating strong C–H bonds. Complex **2** is stable at room temperature and mediates hydrogen atom transfer reactivity via a p*K*_a_–driven, asynchronous PCET mechanism. Kinetic studies reveal a strong correlation between reaction rates and substrate p*K*_a_, but a poor correlation with bond dissociation energies, consistent with a PT–dominant transition state. Thermodynamic analysis estimates the O–H bond dissociation free energy of the resulting Ni­(II)–OH_2_ species to be 96.6–100.3 kcal mol^–1^, in agreement with its ability to activate the strong C–H bond in cyclohexane. These findings suggest that Ni­(III)–OH may serve as a key intermediate in Ni­(II)–catalyzed alkane oxidation reactions. Further mechanistic analysis using semiempirical model developed by Barman et al. yielded a low asynchronicity coefficient (*x* = 0.18), quantitatively supporting a highly proton-dominated asynchronous PCET pathway. The high basicity of the Ni­(III)–OH unit compensates for its low redox potential, enabling oxidative C–H activation of substrates such as toluene and cyclohexane under mild conditions. This reactivity mirrors the strategy employed by cytochrome P450 enzymes where the basicity of ferryl oxo species offsets low redox potential to prevent undesired side reactions.^48^ Collectively, these results establish a mechanistic framework in which basicity, not redox potential or bond strength alone, can govern C–H bond activation. This work not only deepens our understanding of nickel–mediated oxidation chemistry but also offers a guiding principle for the rational design of oxidative catalysts that exploit high basicity to modulate PCET reactivity.

## Supplementary Material



## Notes

Detail descriptions of synthetic procedures and physical methods. Characterization, spectra, and crystallographic data. CCDC 2421982 and 2421983 contain the supplementary crystallographic data of **1**·DCM and [Na­(15c5)]­[**2**]·2THF·H_2_O, respectively (PDF)

## Abbreviations

PS3″: [P­(C_6_H_3_–3–Me_3_Si–2–S)_3_]^3–^
DABCO: 1,4–diazabicyclo­[2.2.2]­octane
HCp*: pentamethylcyclopentadiene
1,4–CHD: 1,4–cyclohexadiene
9,10–DHA: 9,10–dihydroanthrancene,
BAr^F^_4_^–^: [B­(3,5–(CF_3_)_2_–C_6_H_3_)]^−^,
CoCp*_2_: *bis*(η^5^-pentamethylcyclopentadienyl)­cobalt­(II).
