# Supplementary material for: Basicity–Controlled C–H Bond Activation by a Structurally Characterized Ni(III)–Hydroxo Complex
Source: J Am Chem Soc. 2025 Jul 25;147(31):27855–61. doi: 10.1021/jacs.5c06941 (PMC12333028; doi:10.1021/jacs.5c06941)
Supplement: Supplementary file 1 [file ja5c06941_si_001.pdf]

Supporting Information

Basicity−Controlled C−H Bond Activation by a Structurally Characterized Ni(III)−Hydroxo Complex

Hung−Ruei Pan, John Wu, Chun−Ming Tsai, Pei−Juan Liao and Hua−Fen Hsu*

Department of Chemistry, National Cheng Kung University, Tainan 701, Taiwan

General Methods and Materials

All manipulations were conducted under a nitrogen atmosphere using standard Schlenk techniques or in a nitrogen−filled glovebox. The compounds H_3[PS3″] ($H_3[P(C_6H_3-3-Me_3Si-2-S)_3]$) and $[Ni^{III}(PS3'')]_2$ were synthesized according to published procedures.[1, 2] Solvents were dried and distilled by standard methods. All other reagents were purchased from commercial sources and used without further purification unless otherwise noted.

Single crystals were mounted on glass fibers and coated with epoxy resin. X−ray diffraction data were collected using a Bruker D8 Venture diffractometer equipped with an Oxford Cryosystems cooling device. Data processing included absorption corrections using the SADABS program.[3] Structures were solved and refined using the SHELXTL software package.[4] All non−hydrogen atoms were refined anisotropically. Hydrogen atoms were placed in calculated positions using the riding model, except for the hydroxo proton (H1) in [Na(15c5)][**2**]·2THF·H_2O, which was located in the difference Fourier map. UV−vis−NIR spectra were recorded on a Hewlett−Packard 8453 spectrophotometer. NMR spectra (^{1}H, ^{31}P, and ^{1}H ROESY) were obtained on a Bruker AMX500 spectrometer. ^{1}H NMR chemical shifts were referenced to residual solvent peaks, and ^{31}P NMR spectra were referenced to an external standard of PPh_3 (−6.0 ppm). FT−IR spectra were collected using a PerkinElmer Spectrum RX I spectrometer. Elemental analyses were performed by Elementar vario EL III. X−band EPR spectra were collected at 77 K using a Bruker EMX spectrometer, and simulations were carried out using the EasySpin software package. Ni K−edge X−ray absorption spectra were recorded in transmission mode at the BL−17C Wiggler beamline (NSRRC, Taiwan) using a Si(111) double−crystal monochromator. Samples were prepared by grinding single crystals to powder and sealing them in aluminum spacers with Kapton tape under nitrogen. Spectra were collected over an energy range of 8133.0 to 8881.1 eV, with a Ni foil used as an internal energy reference. Incident (I_0) and transmittance (I_t) intensities were recorded using ionization chambers. GC−MS spectra were obtained using a Shimadzu GCMS−QP2010 SE instrument.

Syntheses

[Ni^{III}(PS3″)(DABCO)] (1)

Treatment of [Ni^{III}(PS3″)]$_2$ (0.109 g, 0.087 mmol) with DABCO (2.44 g, 21.7 mmol, 250 equiv.) in THF at ambient temperature for 7 days resulted in a color change from yellow−green to bright green. The solvent was removed under reduced pressure, and the resulting residue was redissolved in CH_2Cl_2 and layered with methanol. After standing at room temperature for 7 days, bright green crystals of **1**·CH_2Cl_2 were obtained in 72% yield. Anal. Calcd for $C_{34}H_{50}Cl_2N_2NiPS_3Si_3$ (**1**·CH_2Cl_2): C, 49.33; N, 3.38; H, 6.09; S, 11.62. Found: C, 49.59; N, 5.77; H, 5.77; S, 11.12. Electronic absorption spectrum in THF (λ, nm; ε, M^{-1} cm^{-1}): 615 (2.67×10^3), 734 (2.0×10^3), and 1023 (1.1×10^3). ^{1}H NMR (CD_2Cl_2): δ 16.75 ppm (s, 3H, Ph), δ 11.95 ppm (bs, 6H, DABCO), δ 10.33 ppm (s, 3H), δ 7.47 ppm (s, 6H, DABCO), δ 1.91 ppm (s, 27H, $SiMe_3$), δ −8.25 ppm (s, 3H, Ph).

[Na(15c5)][Ni^{III}(PS3″)(OH)] ([Na(15c5)][2])

Complex **1** (0.064 g, 0.087 mmol) was treated with sodium hydroxide (0.010 g, 0.26 mmol, 3 equiv.) and 15−crown−5 ether (0.057 g, 0.26 mmol, 3 equiv.) in THF. The reaction mixture was stirred at ambient temperature for 2 days, during which the solution color changed from bright green to dark green. The resulting solution was layered with hexane and stored at −40 °C. After 7 days, dark green crystals of [Na(15c5)][**2**]·2THF·H_2O were isolated in 63% yield. Anal. Calcd for $C_{37}H_{57}NaNiO_6PS_3Si_3$ (**2**): C, 49.88; H, 6.45; S, 10.80. Found: C, 48.41; H, 6.13; S, 10.00. Electronic absorption spectrum in DMSO (λ, nm; ε, M^{-1} cm^{-1}): 585 (2.2×10^3), 734 (1.3×10^3). ^{1}H NMR in d_6−DMSO: δ 15.12 ppm (s, 3H, Ph), δ 8.95 ppm (s, 3H, Ph), δ 3.51 ppm (s, 20H, 15−crown−5), δ 2.55 ppm (s, 27H, $-SiMe_3$), δ −4.01 ppm (s, 3H, Ph).

Kinetic Studies of [Na(15c5)][2] with PCET substrates

Kinetic measurements were carried out using DMSO solutions of [Na(15c5)][**2**] (0.600 mM for all substrates except fluorene, which was measured at 0.300 mM) in quartz cuvettes. The reaction mixtures were equilibrated at 35 °C using a cryostat for approximately 10 minutes before substrate addition. Upon addition of various concentrations of C−H substrates, time−dependent spectral changes were observed. Substrate concentration ranges were as follows: fluorene (2.80−7.00 mM), HCp* (124−309 mM), 1,4−CHD (21.1−106 mM), xanthene (28.1−314 mM), and 9,10−DHA (28.1−281 mM). Reactions were monitored by the exponential decay of absorbance at 585 nm. Pseudo−first−order rate constants (k_{obs}) were determined by fitting the absorbance−time profiles to the following equation. Typically, three half−lives of the decay were used for the fitting.[5]

$$Abs_t = Abs_f + (Abs_0 - Abs_f) \times e^{-k_{obs}t}$$

where Abs_t is the absorbance at time t, and Abs_0 and Abs_f are the initial and final absorbance values at 585 nm, respectively. Second−order rate constants (k_2) were derived from the slope of the linear plot of k_{obs} versus the concentration of the corresponding substrate.

Table S1. Crystallographic data of $[Ni^{III}(PS3'')(DABCO)]\cdot CH_2Cl_2$ ($\mathbf{1}\cdot CH_2Cl_2$)

Empirical formula	$C_{34}H_{50}Cl_2N_2NiPS_3Si_3$	
Formula weight	827.79	
Crystal system	Monoclinic	
Space group	$P2_1/c$	
Temperature	100(2) K	
Unit cell dimensions	a = 14.8860(7) Å	$\alpha = 90^\circ$
	b =11.6194(5) Å	$\beta = 92.3120(10)^\circ$
	c = 23.0558(11) Å	$\gamma = 90^\circ$
Volume	3984.6(3) $Å^3$	
Z	4	
Density (calculated)	1.380 Mg/m^3	
Absorption coefficient	0.936 mm^{-1}	
F(000)	1740	
Reflections collected	82257	
Independent reflections	9913 [R(int) = 0.0265]	
GOF[c]	1.119	
R_1[a] [I>2sigma(I)]	0.0277	
wR_2[b] [I>2sigma(I)]	0.0677	

[a]$GOF=\{\Sigma[w(F_o-F_c^2)^2]/(M-N)\}^{1/2}$ (M= number of reflections, N= number of parameters refined)

[b]$R_1= \Sigma||F_o|-|F_c||/\Sigma|F_o|$

[c]$wR_2= \{\Sigma[w(F_o^2-F_c^2)^2]/\Sigma[w(F_o^2)^2]\}^{1/2}$

Table S2. Selected bond lengths and angles of $[Ni^{III}(PS3'')(DABCO)]\cdot CH_2Cl_2$ (**1**$\cdot CH_2Cl_2$)

Bond length (Å)	
Ni(1)–N(1)	2.033(1)
Ni(1)–P(1)	2.1238(4)
Ni(1)–S(1)	2.2285(4)
Ni(1)–S(2)	2.2674(4)
Ni(1)–S(3)	2.2652(4)
Bond angle (degree)	
P(1)–Ni(1)–N(1)	178.98(3)
P(1)–Ni(1)–S(1)	86.10(1)
P(1)–Ni(1)–S(2)	84.66(1)
P(1)–Ni(1)–S(3)	83.85(1)
N(1)–Ni(1)–S(1)	94.87(3)
N(1)–Ni(1)–S(2)	95.13(3)
N(1)–Ni(1)–S(3)	95.33(3)
S(1)–Ni(1)–S(2)	118.86(2)
S(2)–Ni(1)–S(3)	112.74(2)
S(1)–Ni(1)–S(3)	126.03(2)

Table S3. Crystallographic data of [Na(15c5)][Ni^{III}(PS3″)(OH)]·2THF·0.5H_2O ([Na(15c5)][**2**]·2THF·0.5H_2O)

Empirical formula	$C_{45}H_{73}NaNiO_{8.50}PS_3Si_3$	
Formula weight	1043.15	
Crystal system	Triclinic	
Space group	P$\bar{1}$	
Temperature	100(2) K	
Unit cell dimensions	a = 11.7321(6) Å	α = 81.8428(16)°
	b =13.3073(7) Å	β = 73.6320(15)°
	c = 19.8417(11) Å	γ = 79.9972(16)°
Volume	2912.8(3) Å^3	
Z	2	
Density (calculated)	1.189 Mg/m^3	
Absorption coefficient	0.581 mm^{-1}	
F(000)	1110	
Reflections collected	43804	
Independent reflections	10280 [R(int) = 0.0215]	
GOF^c	1.169	
R_1^a [I>2sigma(I)]	0.0533	
wR_2^b [I>2sigma(I)]	0.1523	

^{a}GOF=$\{\Sigma[w(F_o{-}F_c^2)^2]/(M{-}N)\}^{1/2}$ (M= number of reflections, N= number of parameters refined)

$^b R_1 = \Sigma||F_o|-|F_c||/\Sigma|F_o|$

$^c wR_2 = \{\Sigma[w(F_o^2-F_c^2)^2]/\Sigma[w(F_o^2)^2]\}^{1/2}$

Table S4. Selected bond lengths and angles of [Na(15c5)][Ni^{III}(PS3″)(OH)]·2THF·0.5H_2O ([Na(15c5)][**2**]·2THF·0.5H_2O)

Bond length (Å)	
Ni(1)−O(1)	1.891(2)
Ni(1)−P(1)	2.107(1)
Ni(1)−S(1)	2.2907(8)
Ni(1)−S(2)	2.3101(8)
Ni(1)−S(3)	2.2359(8)
Bond angle (degree)	
P(1)−Ni(1)−O(1)	174.57(7)
P(1)−Ni(1)−S(1)	81.64(3)
P(1)−Ni(1)−S(2)	84.40(3)
P(1)−Ni(1)−S(3)	87.04(3)
O(1)−Ni(1)−S(1)	93.96(7)
O(1)−Ni(1)−S(2)	100.16(7)
O(1)−Ni(1)−S(3)	94.59(7)
S(1)−Ni(1)−S(2)	110.33(3)
S(2)−Ni(1)−S(3)	104.24(3)
S(3)−Ni(1)−S(1)	142.18(3)
H(1)−O(1)−Ni(1)	99(2)
Na(1)−O(1)−Ni(1)	145.4(1)

Table S5. Kinetic data for the Reaction of [Na(15c5)][**2**] with various substrates.

Substrate	Concentration (M)	k_{obs} (s^{-1}) [a]	k_2 ($M^{-1}s^{-1}$)
fluorene	0.0028	$1.76(6)\times10^{-3}$	8.70×10^{-1}
	0.0042	$2.7(2)\times10^{-3}$	
	0.0056	$3.8(1)\times10^{-3}$	
	0.0070	$5.5(3)\times10^{-3}$	
d_{10}−fluroene	0.0045	$8(1)\times10^{-4}$	1.70×10^{-1}
	0.0075	$1.4(3)\times10^{-3}$	
	0.0150	$2.8(5)\times10^{-3}$	
	0.0225	$3.8(3)\times10^{-3}$	
HCp*	0.1235	$2.17(3)\times10^{-3}$	1.92×10^{-2}
	0.1852	$3.14(7)\times10^{-3}$	
	0.2469	$4.7(5)\times10^{-3}$	
	0.3087	$5.6(5)\times10^{-3}$	
1,4−CHD	0.0422	3.02×10^{-4}	7.87×10^{-3} [b]
	0.0528	$4.7(6)\times10^{-4}$	
	0.0704	8.4×10^{-4}	
	0.1056	13.0×10^{-4}	
xanthene	0.0310	$7(7)\times10^{-5}$	1.26×10^{-3}
	0.0630	$1.1(6)\times10^{-4}$	
	0.1260	$1.7(9)\times10^{-4}$	
	0.1890	$2.8(6)\times10^{-4}$	
9,10−DHA	0.0561	$9.1(5)\times10^{-5}$	5.02×10^{-4} [b]
	0.1122	$1.50(7)\times10^{-4}$	
	0.1683	$1.9(1)\times10^{-4}$	
	0.2806	$3.2(3)\times10^{-4}$	

[a] Each experiment was repeated three times.

[b] The k_2 value is half the slope of the k_{obs} versus substrate concentration plots, reflecting the stoichiometry of the two−hydrogen oxidation of 1,4−CHD and 9,10−DHA.[6]

Table S6. Organic products formed from substrate reactions with [Na(15c5)][**2**].

Substrate	Product	Yield (%)
fluorene	d_1−fluroene	78
HCp*	1,1',2,2',3,3',4,4',5,5'−decamethyl−[1,1'−bi(cyclopentane)]−2,2',4,4'−tetraene	~100
1,4−CHD	benzene	~100
xanthene	9,9'−bixanthene	74
9,10−DHA	9,9',10,10'−tetrahydro−9,9'−bianthracyl	25
	anthracene	63

Table S7. Thermodynamic parameters of substrates and corresponding asynchronicity values (η).

Substrate	$pK_a^{R,ox}$ [7, 8]	E^0_{R-} (V, vs. Fc/Fc$^+$) [8, 9]	η
fluorene	−17	−1.069	−2.30
HCp*	−6.5	−1.379	−1.63
xanthene	−18	−1.685	−1.91
9,10−DHA	−24	−1.575	−2.24

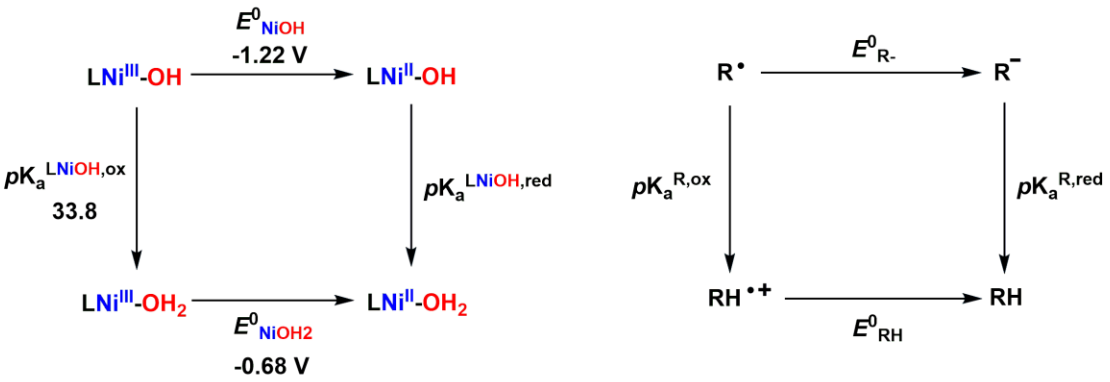

The asynchronicity values (η) were calculated using Equations S1−S3 following the method reported:[10]

$$\eta = 2^{-\frac{1}{2}} \times \left[\Delta E^0 - \left(\frac{RT}{F}\right) \times ln10 \times \Delta pK_{a,ox}\right] \; (Eq.S1)$$

$$\Delta E^0 = E^0_{NiOH} - E^0_{R-} \; (Eq.S2)$$

$$\Delta pK_{a,ox} = pK_a{}^{Ni(III)OH,ox} - pK_a{}^{R,ox} \; (Eq.S3)$$

As an example, for 9,10−DHA, the ΔE^0 and $\Delta pK_{a,ox}$ values were calculated from the tabulated thermodynamic data of complex **2** and 9,10−DHA, as shown in Equations S4 and S5:

$$\Delta E^0 = (-1.22) - (-1.575) = 0.355 \text{ V} \; (Eq.S4)$$

$$\Delta pK_{a,ox} = (33.8) - (-24) = 57.8 \; (Eq.S5)$$

The value of η was subsequently calculated from ΔE^0 and $\Delta pK_{a,ox}$ using Equation S6:

$$\eta = 2^{-\frac{1}{2}} \times \left[0.355 - \left(\frac{RT}{F}\right) \times ln10 \times 57.8\right] = -2.24 \; (Eq.S6)$$

Table S8. Thermodynamic parameters of substrates and the corresponding ΔG^0_{PT} and ΔG^0_{ET} values used in semiempirical model of Braman *et al.*

Substrate	$pK_a^{R,red}$ [9, 11]	E^0_{R-} (V, vs. Fc/Fc^+) [8, 9]	ΔG^0_{PT} (kcal mol^{-1})	ΔG^0_{ET} (kcal mol^{-1})
fluorene	22.6	−1.069	−15.09	−8.97
HCp*	26.1	−1.379	−10.38	−16.12
xanthene	30.0	−1.685	−5.12	−23.18
9,10−DHA	30.1	−1.575	−4.99	−20.64

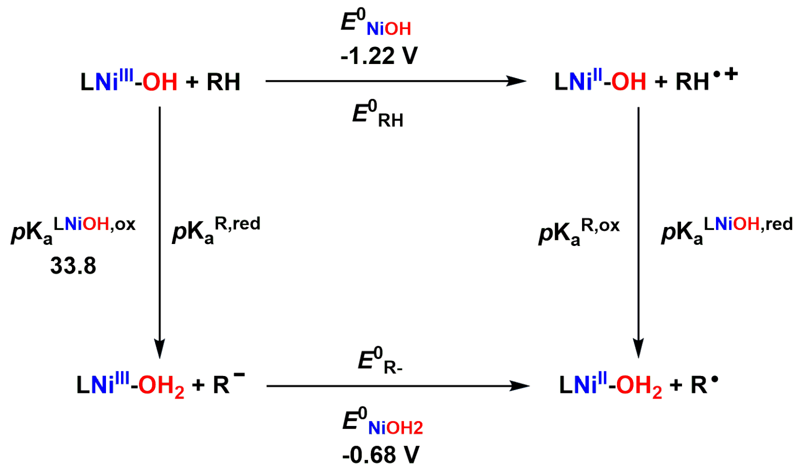

To evaluate the asynchronicity of PCET reactivity for complex **2** using the model of Braman *et al*, Equations S7−S9 were applied:[12]

$$log\, k_2 = -\left(\frac{\alpha}{2.303RT}\right)(\Delta G^0_{PT} + x\Delta G^0_{ET}) + \beta' \; (Eq.S7)$$

$$\Delta G^0_{PT} = -2.303RT(pK_a^{LNiOH,ox} - pK_a^{R,red}) \; (Eq.S8)$$

$$\Delta G^0_{ET} = -F(E^0_{NiOH2} - E^0_{R-}) \; (Eq.S9)$$

As an example, for 9,10−DHA, the ΔG^0_{PT} and ΔG^0_{ET} values were calculated from the tabulated thermodynamic data of complex **2** and 9,10−DHA, as shown in Equations S10 and S11:

$$\Delta G^0_{PT} = -2.303 \times 0.0019 \times 308 \times (33.8 - 30.1) = -4.99\; (kcal\; mol^{-1})\; (Eq.S10)$$

$$\Delta G^0_{ET} = -23.06 \times [(-0.68) - (-1.575)] = -20.64\; (kcal\; mol^{-1})\; (Eq.S11)$$

The correlation of $(\Delta G^0_{PT} + x\Delta G^0_{ET})$ against logk_2 was plotted by adjusting x value from 1 to 0. The strongest correlation occurred x= 0.18 with an R^2 value of 0.99 as shown in Figure S27.

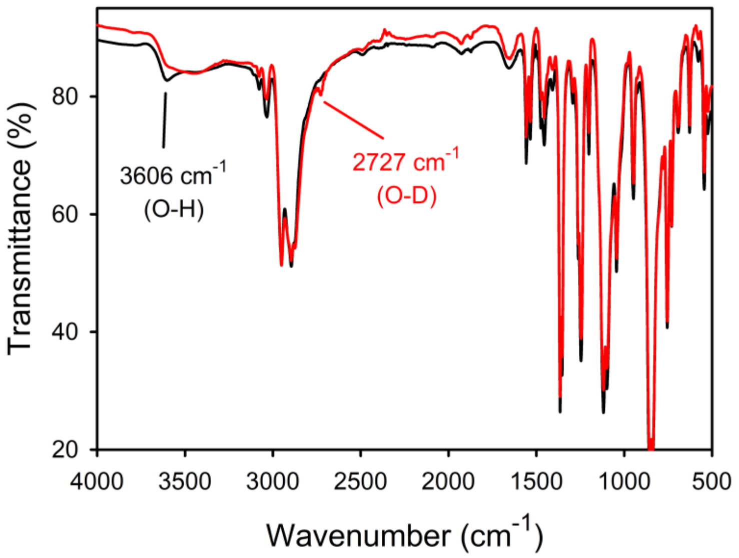

Figure S1. IR spectra of [Na(15c5)][Ni^{III}(PS3″)(OH)] (black) and [Na(15c5)][Ni^{III}(PS3″)(OD)] (red) recorded in KBr.

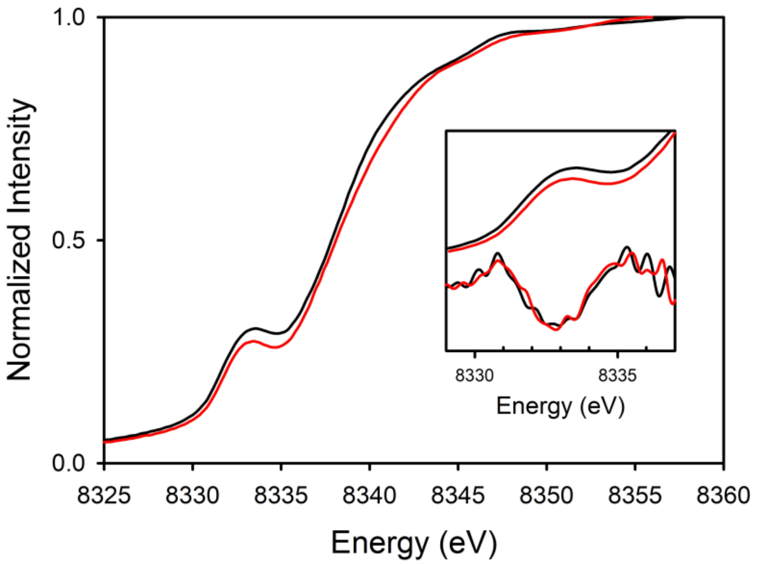

Figure S2. Normalized Ni K−edge X−ray absorption spectra of **1** (black) and [Na(15c5)][**2**] (red). Inset: expanded view of the pre−edge region (top) and corresponding second derivative spectra (bottom).

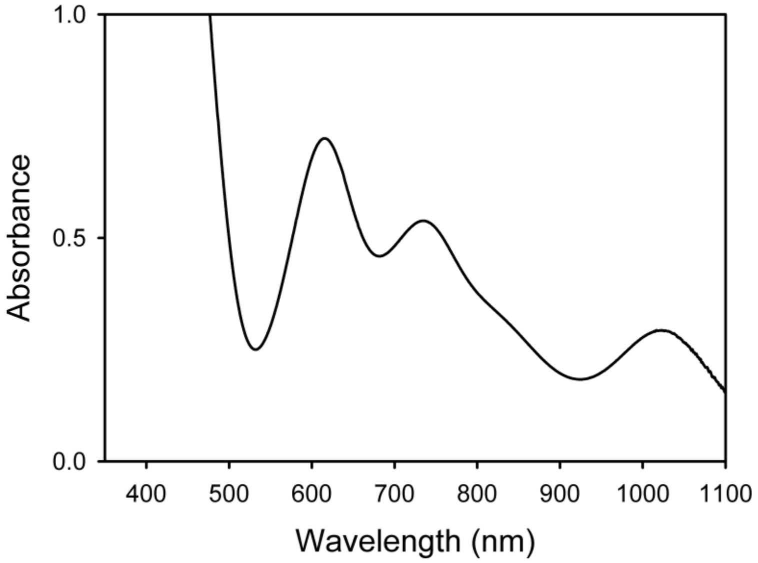

Figure S3. UV−vis−NIR spectrum of complex **1** (0.22 mM) in THF.

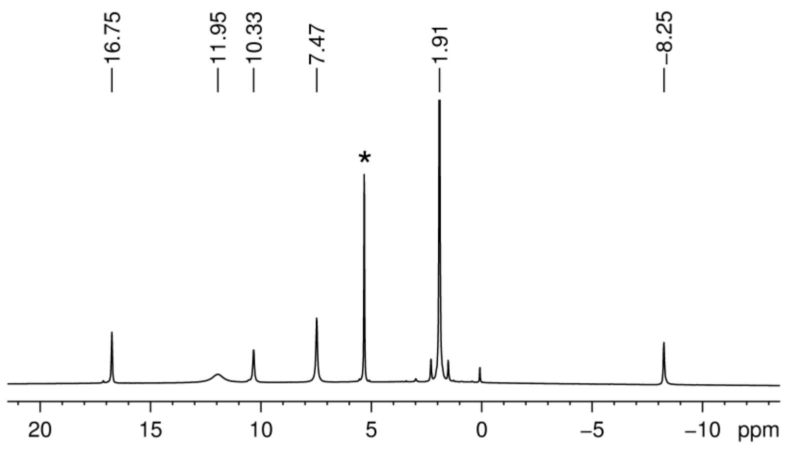

Figure S4. ^{1}H NMR spectrum of complex **1** in CD_2Cl_2. Asterisk indicates the residual solvent signal.

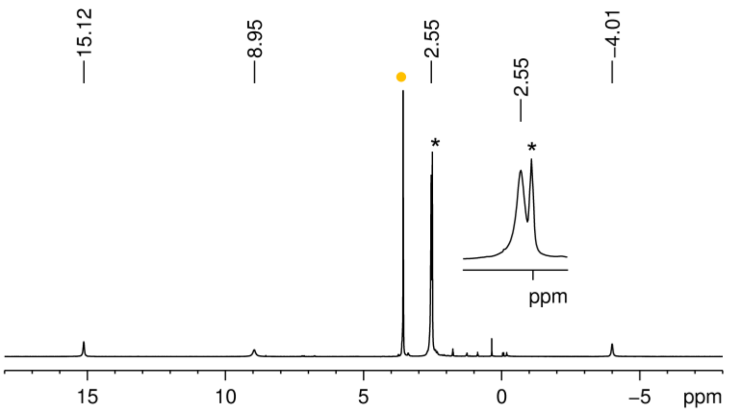

Figure S5. ^{1}H NMR spectrum of [Na(15c5)][**2**] in d_6–DMSO. Asterisk indicates the residual solvent signal.; ●: [Na(15c5)]$^+$.

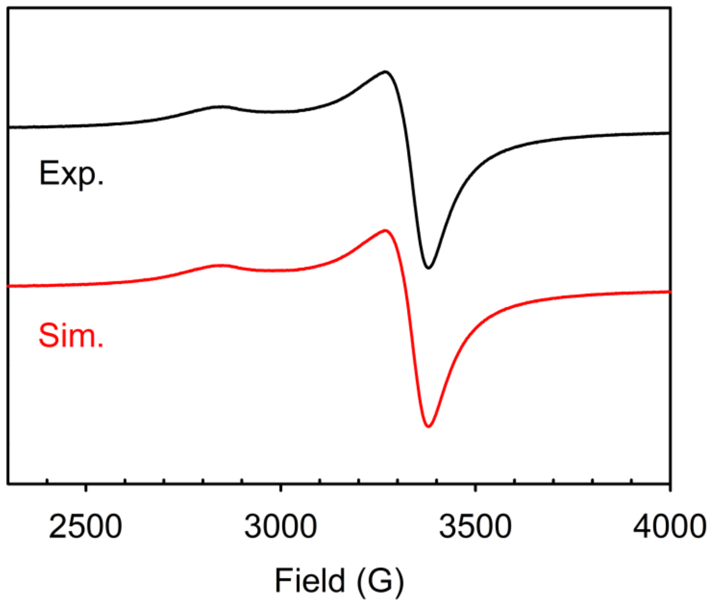

Figure S6. X−band EPR spectrum of complex **1** in 2−MeTHF (5 mM) at 77 K. Sweep range: 2300−4000 G; microwave frequency: 9.36 GHz; modulation amplitude: 0.00016 T; modulation frequency: 100 kHz; microwave power: 3.17 mW. Simulated spectrum best fit using g values of 2.40, 2.04, and 2.01.

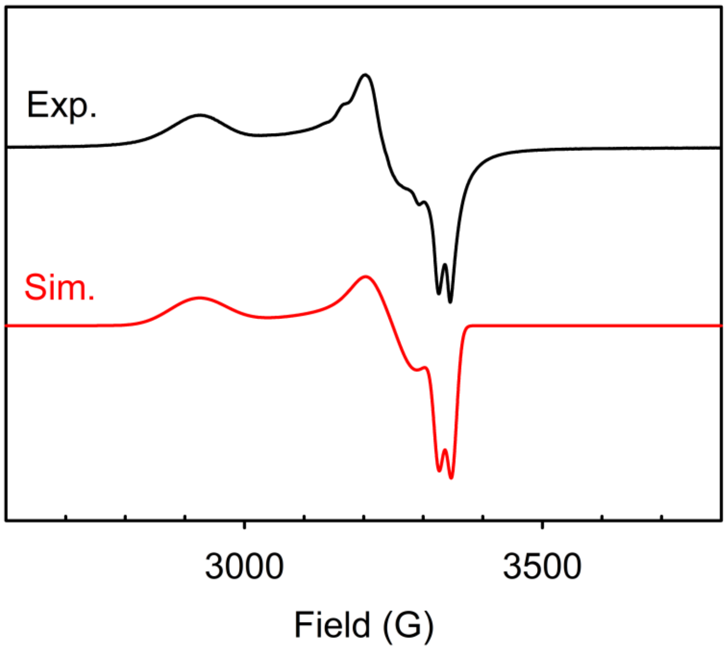

Figure S7. X−band EPR spectrum of [Na(15c5)][**2**] in 2−MeTHF (3 mM) at 77 K. Sweep range: 2600−3800 G; microwave frequency: 9.36 GHz; modulation amplitude: 0.00016 T; modulation frequency: 100 kHz; power: 0.63 mW. Best−fit simulation parameters: *g* (2.33, 2.09, 2.04), A_P (140, 110, 60) × 10^{-4} cm^{-1}.

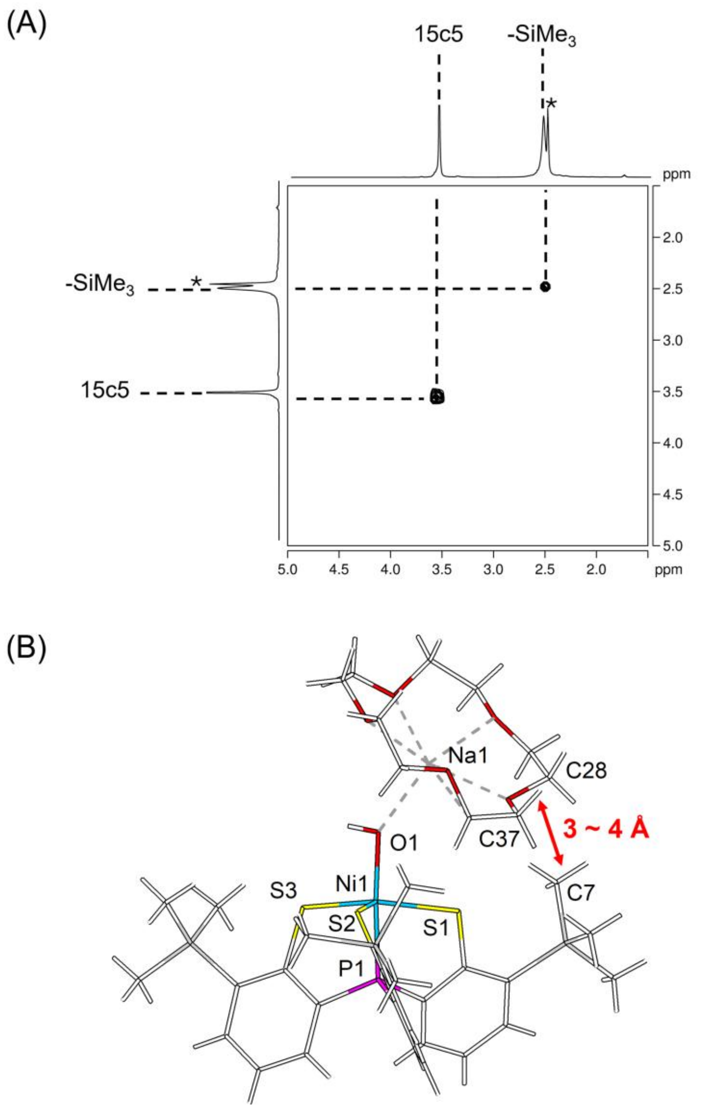

Figure S8. (A) ^{1}H ROESY NMR spectrum of [Na(15c5)][**2**] in d_6−DMSO showing the region from 5.0 to 1.5 ppm. (B) Solid−state structure highlighting the distance between the 15−crown−5 ether and the trimethylsilyl group in the crystal lattice.

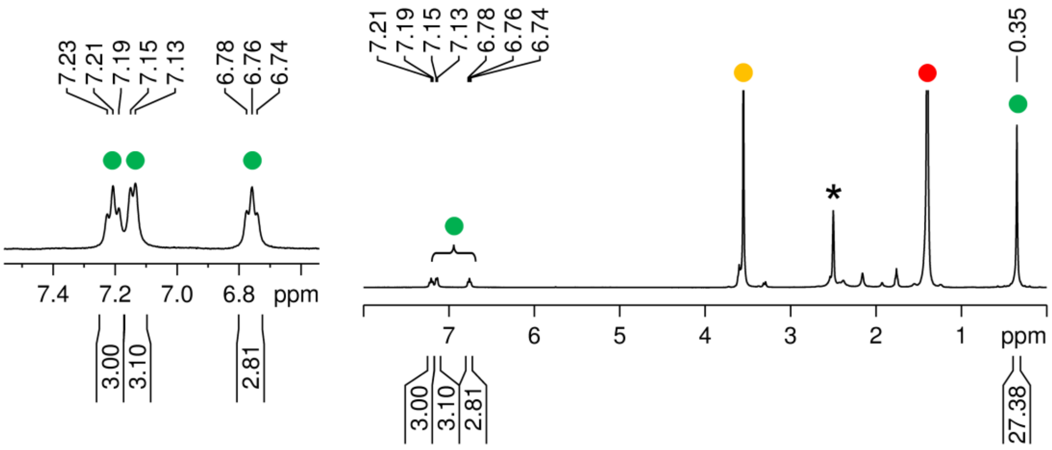

Figure S9. ^{1}H NMR spectrum of [Na(15c5)][**2**] (15mM) and 25 equiv. of cyclohexane in d_6–DMSO after 5 days at room temperature. *: d_6–DMSO; ●: **3**; ●: [Na(15c5)]$^+$; ●: cyclohexane.

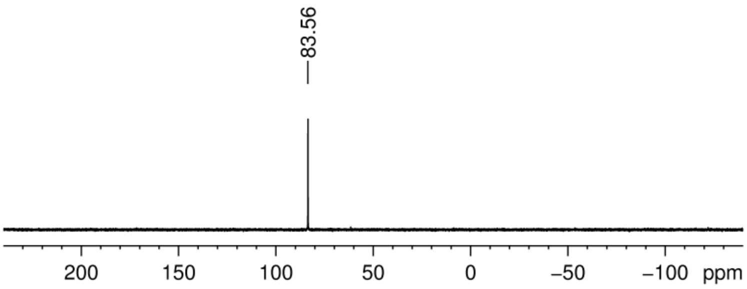

Figure S10. ^{31}P NMR spectrum of [Na(15c5)][**2**] (15mM) and 25 equiv. of cyclohexane in d_6−DMSO after 5 days at room temperature.

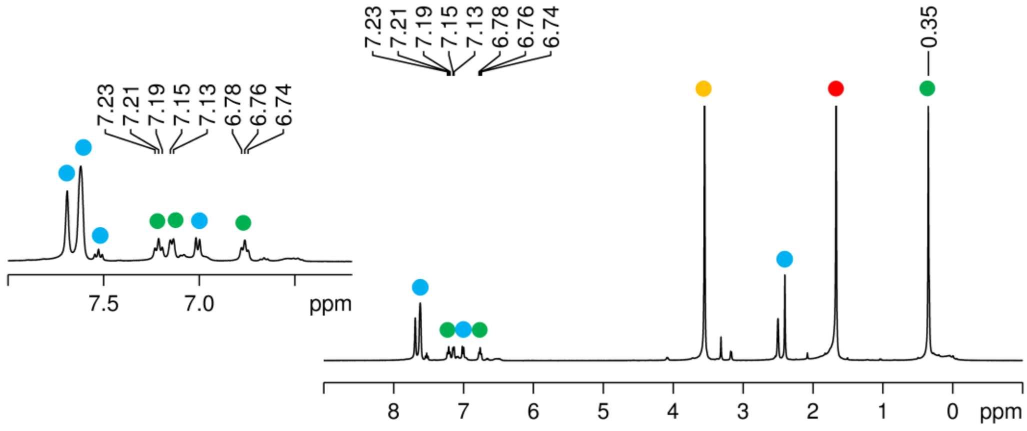

Figure S11. ^{1}H NMR spectrum of [Na(15c5)][**2**] after reaction with 1 equiv. each of [2,6−LutH][BAr^F$_4$] and CoCp*2 in d_6−DMSO. *: d_6−DMSO; ●: **3**; ●: [Na(15c5)]$^+$; ●: 2,6−Lut[BAr^F$_4$$^-$]; ●: CoCp*$_2$$^+$.

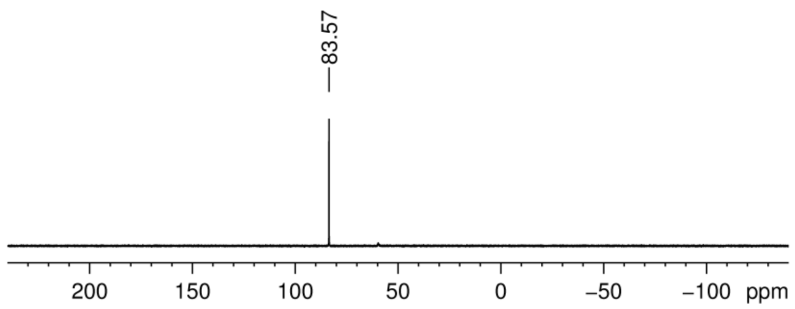

Figure S12. ^{31}P NMR spectrum of [Na(15c5)][**2**] after reaction with 1 equiv. each of [2,6−LutH][BAr^F_4] and $CoCp^*_2$ in d_6−DMSO.

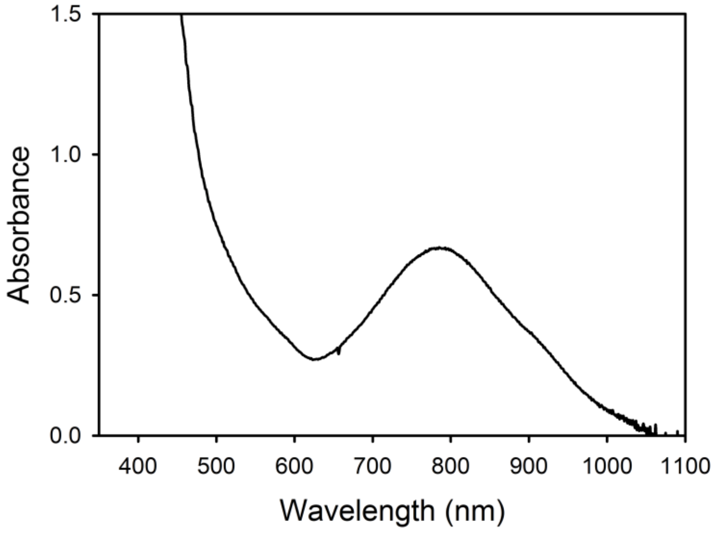

Figure S13. UV−vis−NIR spectrum of the post−reaction solution of [Na(15c5)][**2**] (0.6 mM) with 200 equiv. of toluene in DMSO.

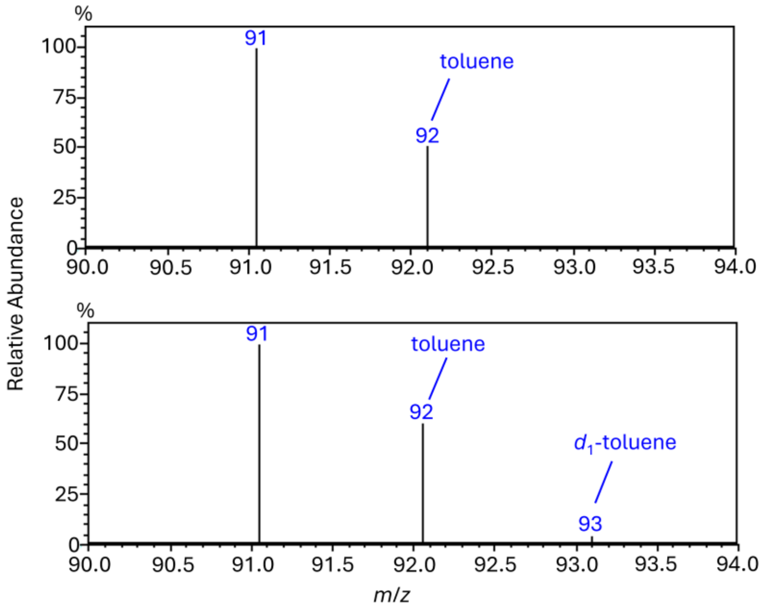

Figure S14. GC−MS spectrum of post−reaction solution of [Na(15c5)][**2**] and toluene in (top) h_6−DMSO and (bottom) d_6−DMSO.

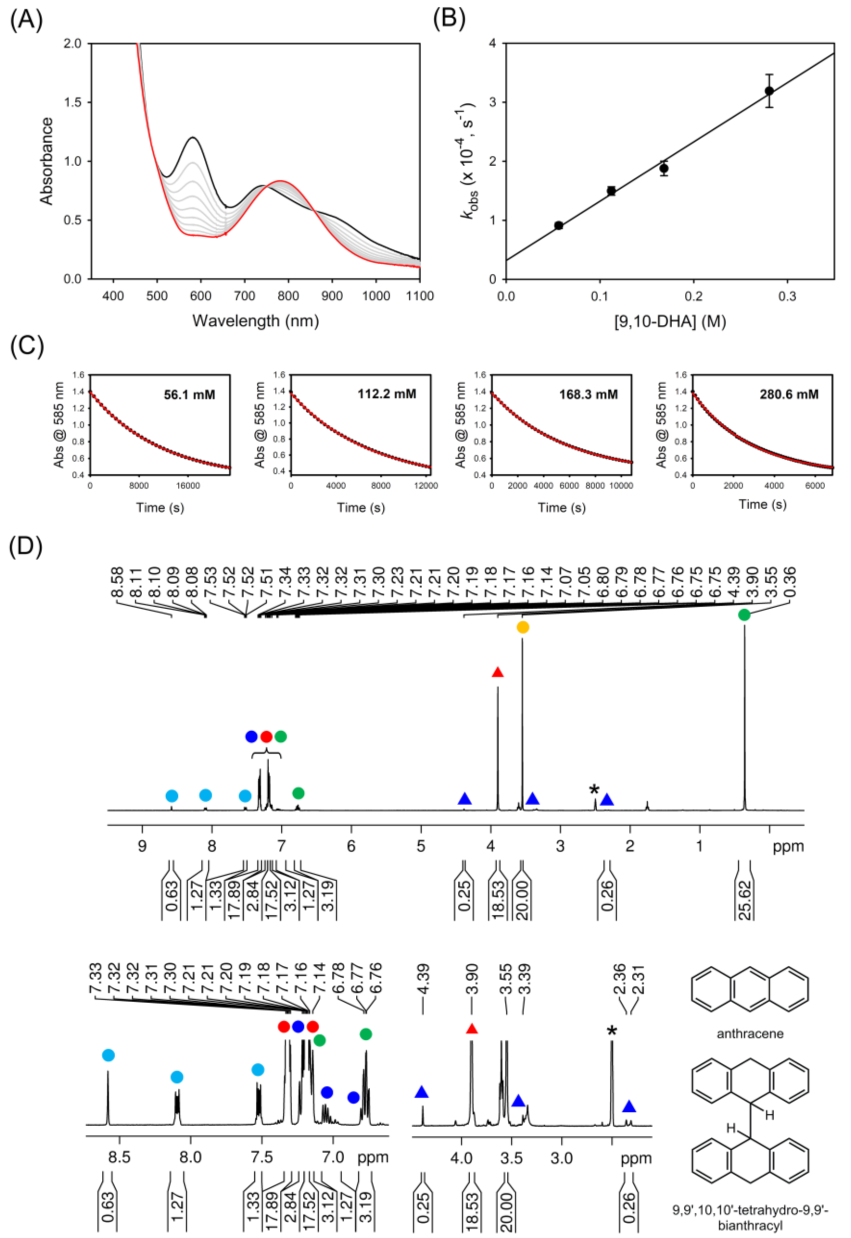

Figure S15. (A) UV−vis−NIR spectral change of [Na(15c5)][**2**] (0.6 mM) upon reaction with 9,10−DHA (0.0561 M) in DMSO at 35 °C. Initial: black line; final: red line. (B) Plot of k_{obs} versus the concentration of 9,10−DHA. (C) Time−dependent absorbance plots at 585 nm with varying concentrations. Black dot: experimental data; red line: nonlinear least squares fit to an exponential decay. (D) ^{1}H NMR spectrum of [Na(15c5)][**2**] with 5.5 equiv. 9,10−DHA in d_6−DMSO with expanded view of selected regions. (*: d_6−DMSO; ●: **3**; ●: [Na(15c5)]$^+$; ●: aromatic H in 9,9',10,10'−tetrahydro−9,9'−bianthryl; ▲: nonaromatic H in 9,9',10,10'−tetrahydro−9,9'−bianthracyl; ●: aromatic H in anthracene; ●: aromatic H in 9,10−DHA; ▲: H of methylene in 9,10−DHA)

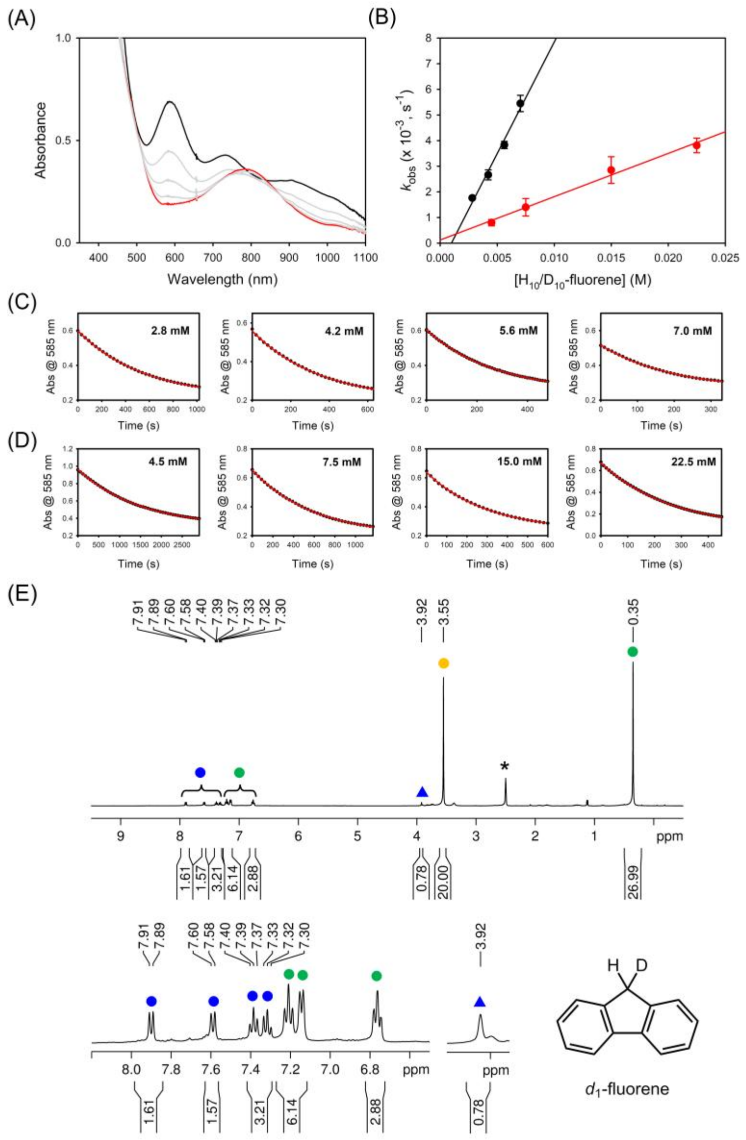

Figure S16. (A) UV−vis−NIR spectral change of [Na(15c5)][**2**] (0.3 mM) upon reaction with fluorene (0.0028 M) in DMSO at 35 ºC. Initial: black line; final: red line. (B) Plot of k_{obs} versus the concentration of fluorene (black) and d_{10}−fluroene (red). (C) Time−dependent absorbance plots at 585 nm with varying concentrations of fluorene. (D) Time−dependent absorbance plots at 585 nm with varying concentrations of d_{10}−fluroene. Black dot: experimental data; red line: nonlinear least squares fit to an exponential decay. (E) ^1H NMR spectrum of [Na(15c5)][**2**] with 1 equiv. fluorene in d_6−DMSO with expanded view of selected regions. (*: d_6−DMSO; ●: **3**; ●: [Na(15c5)]$^+$; ●: aromatic H in fluorene; ▲: H of methylene in fluorene)

Note: The generated fluorenyl radical captured a D atom from the deuterated solvent, resulting in the observation of d_1−fluorene in the ^1H NMR spectrum. The same observation was reported in the literature.[12]

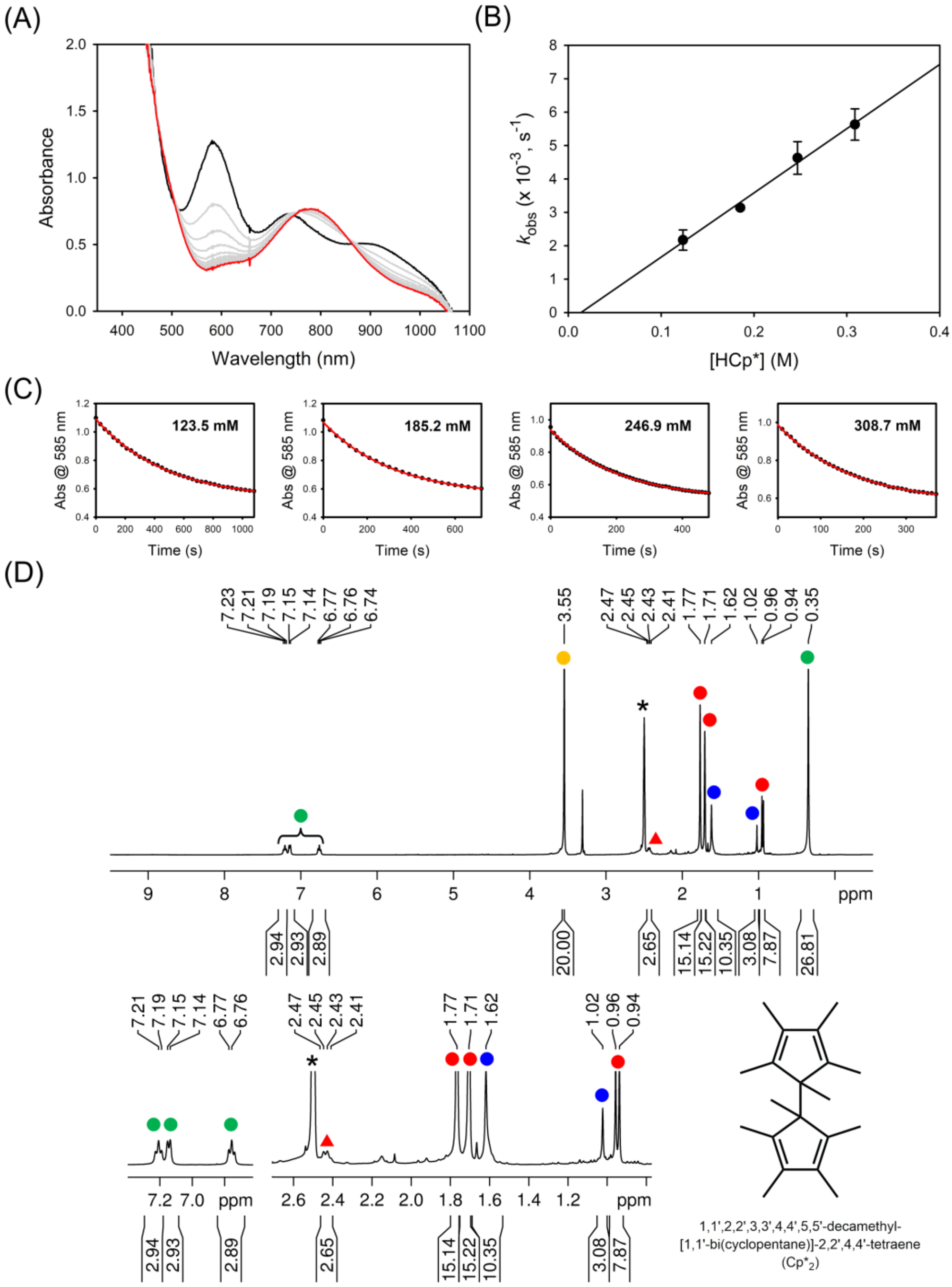

Figure S17. (A) UV−vis−NIR spectral change of [Na(15c5)][**2**] (0.6 mM) upon reaction with HCp* (0.1235 M) in DMSO at 35 °C. Initial: black line; final: red line. (B) Plot of k_{obs} versus the concentration of HCp*. (C) Time−dependent absorbance plots at 585 nm with varying concentrations. Black dot: experimental data; red line: nonlinear least squares fit to an exponential decay. (D) ^{1}H NMR spectrum of [Na(15c5)][**2**] with 4.5 equiv. HCp* in d_6−DMSO with expanded view of selected regions. (*: d_6−DMSO; ●: **3**; ●: [Na(15c5)]$^+$; ●: H in Cp*$_2$; ●: H of methyl groups in HCp*; ▲: H of tertiary C in HCp*)

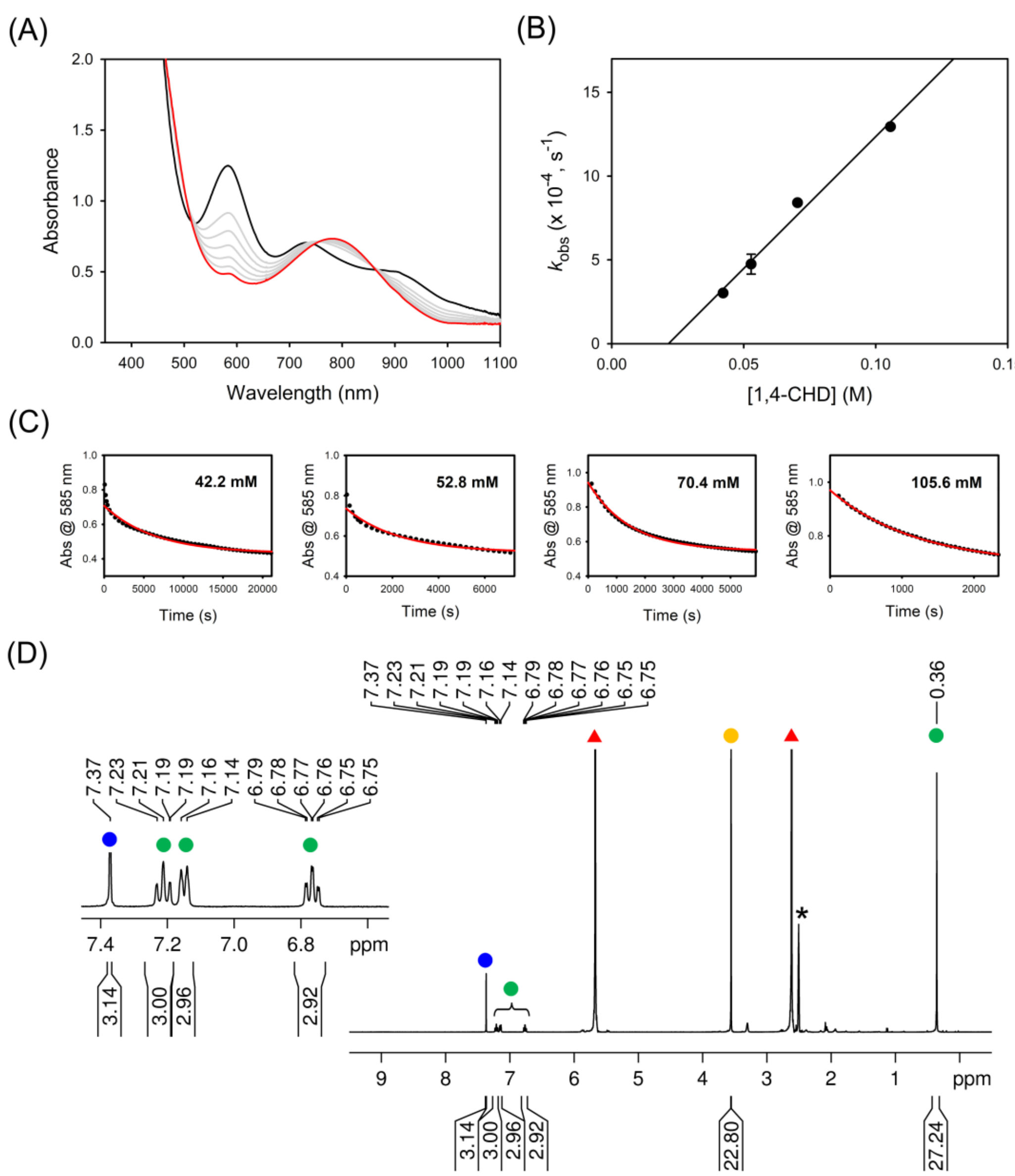

Figure S18. (A) UV−vis−NIR spectral change of [Na(15c5)][**2**] (0.6 mM) upon reaction with 1,4−CHD (0.0352 M) in DMSO at 35 °C. Initial: black line; final: red line. (B) Plot of k_{obs} versus the concentration of 1,4−CHD. (C) Time−dependent absorbance plots at 585 nm with varying concentrations. Black dot: experimental data; red line: nonlinear least squares fit to an exponential decay. (D) ^{1}H NMR spectrum of [Na(15c5)][**2**] with 32 equiv. 1,4−CHD in d_6−DMSO with expanded view of selected regions. (*: d_6−DMSO; ●: **3**; ●: [Na(15c5)]$^+$; ●: benzene; ▲: H of 1,4−CHD)

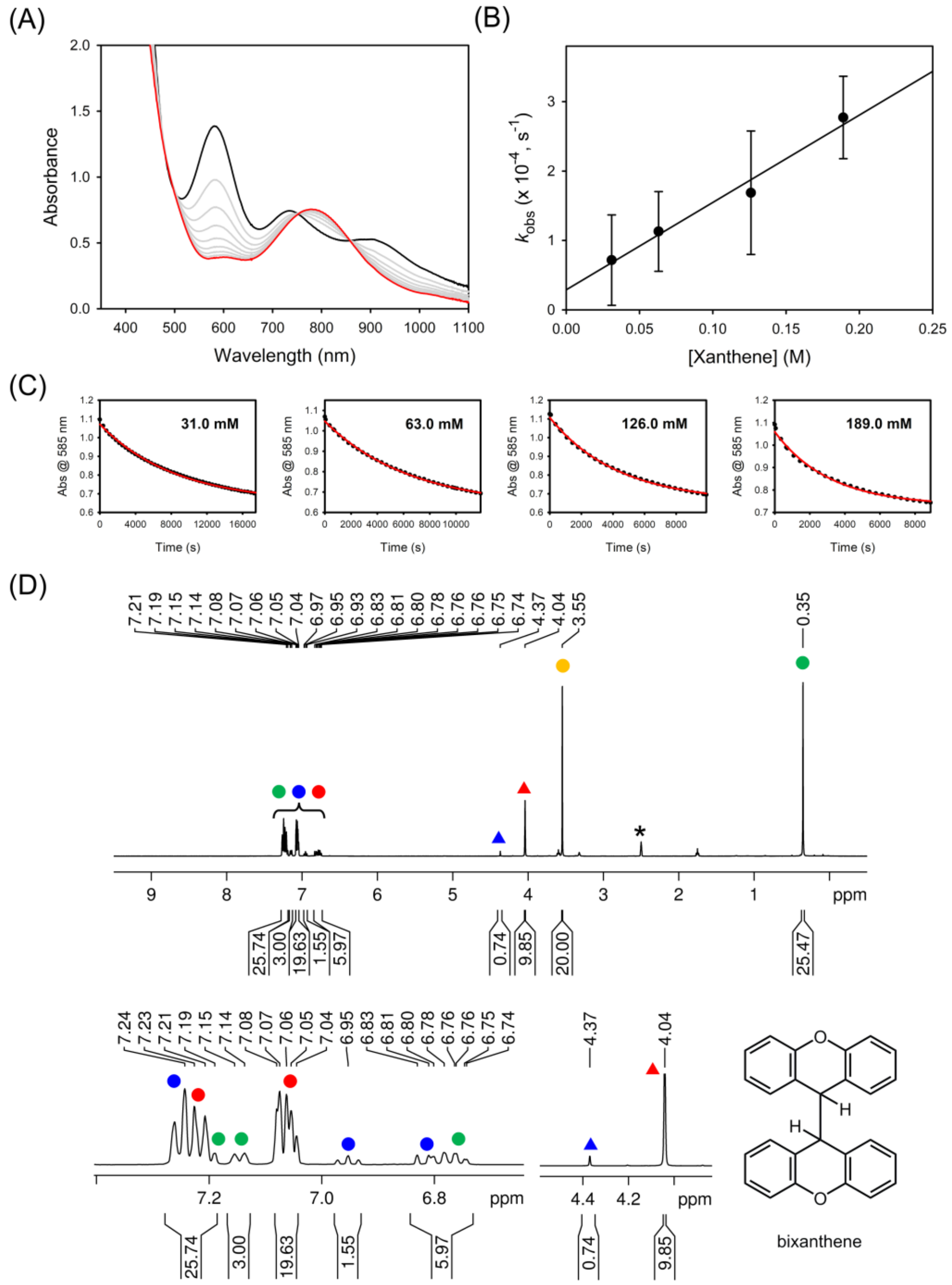

Figure S19. (A) UV−vis−NIR spectral change of [Na(15c5)][**2**] (0.6 mM) upon reaction with xanthene (0.0281 M) in DMSO at 35 °C. Initial: black line; final: red line. (B) Plot of k_{obs} versus the concentration of xanthene. (C) Time−dependent absorbance plots at 585 nm with varying concentrations. Black dot: experimental data; red line: nonlinear least squares fit to an exponential decay. (D) ^{1}H NMR spectrum of [Na(15c5)][**2**] with 5.5 equiv. xanthene in d_6−DMSO with expanded view of selected regions. (*: d_6−DMSO; ●: **3**; ●: [Na(15c5)]$^+$; ●: aromatic H in bixanthene; ▲: nonaromatic H in bixanthene; ●: aromatic H in xanthene; ▲: H of methylene in xanthene)

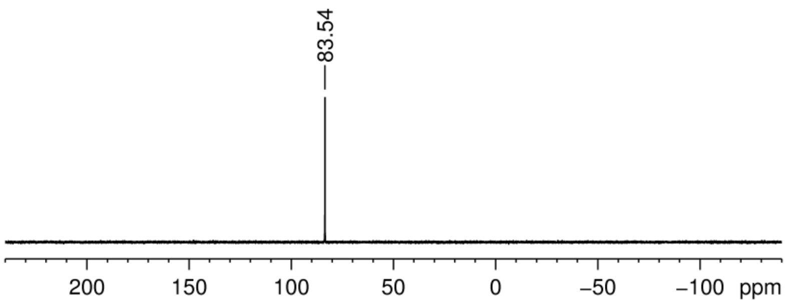

Figure S20. ^{31}P NMR spectrum of [Na(15c5)][**2**] after reaction with 5.5 equiv. of 9,10−DHA in d_6−DMSO.

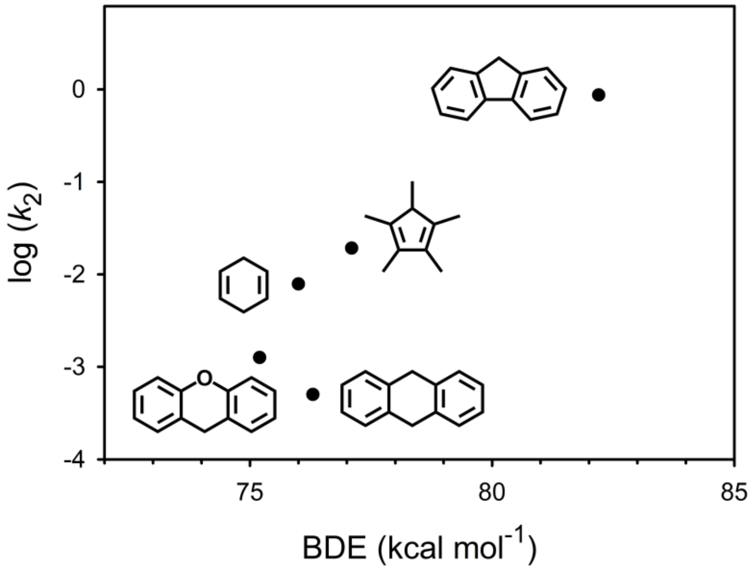

Figure S21. Plot of log (k_2) versus BDE.

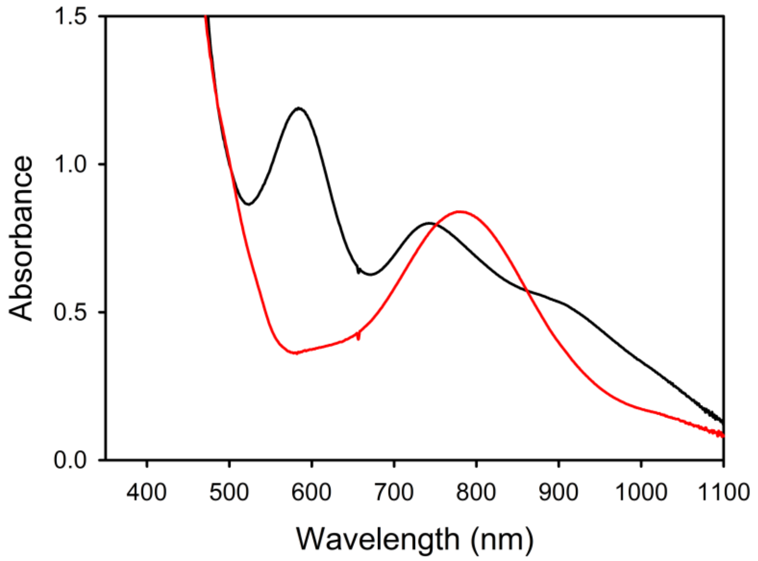

Figure S22. UV−vis−NIR spectra of [Na(15c5)][**2**] (0.6 mM) with indene (0.006 M) in DMSO at 35 ºC. Initial: black line; final: red line.

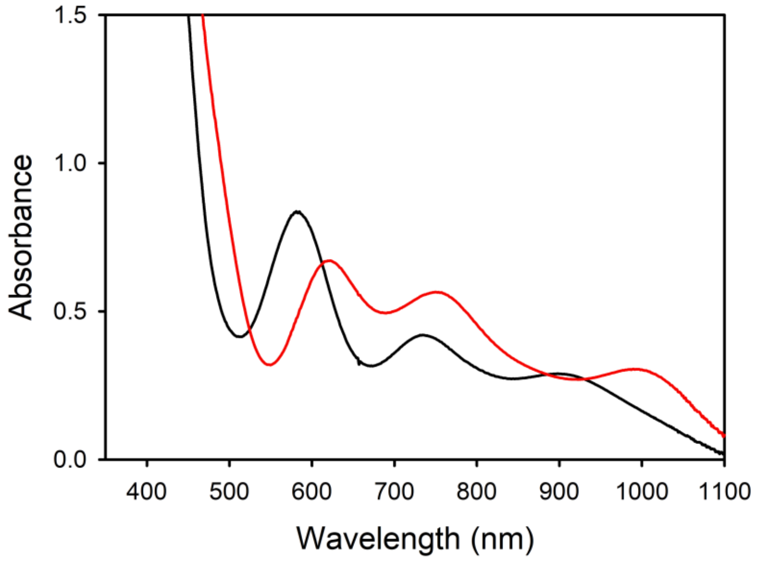

Figure S23. UV−vis−NIR spectra of [Na(15c5)][**2**] in DMSO before (black) and after addition of 1 equiv. of [2,6−LutH][$BAr^F{}_4$] (red).

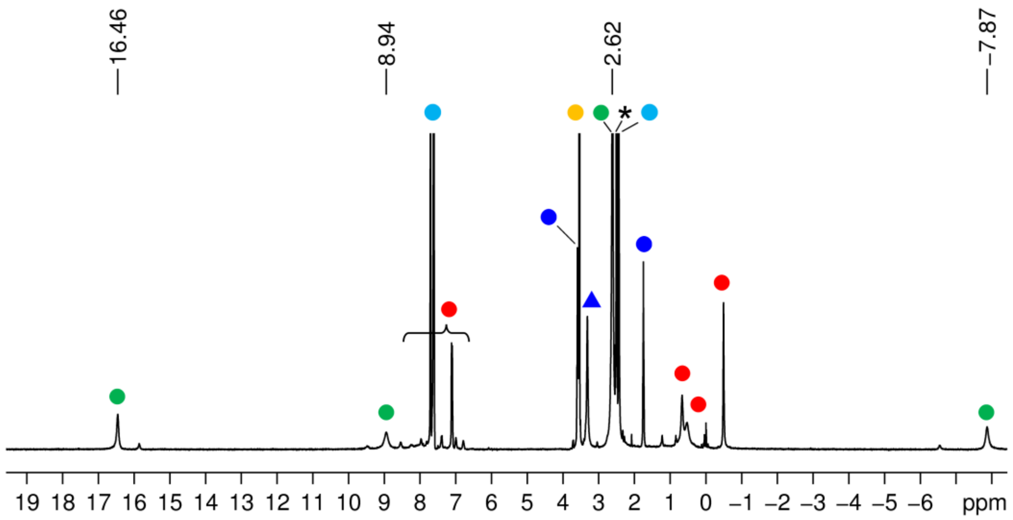

Figure S24. ^{1}H NMR spectrum of [Na(15c5)][**2**] after addition of 1eqiv of [2,6–LutH][BAr^F_4] in d_6–DMSO. *: d_6–DMSO; ●: **2–H**$^+$; ●: $[Na(15c5)]^+$; ●: $LutBAr^F_4{}^-$; ●: $[Ni^{III}(PS3'')]_2$; ●: THF; ▲: H_2O.

Note: The protonated species **2–H**$^+$ underwent dimerization in solution, likely due to the lability of the coordinated H_2O ligand, resulting in the formation of a Ni^{III}/Ni^{III} dimer in less than 30% yield. This species was also detected in the cyclic voltammetry data shown in **Figure S26**.

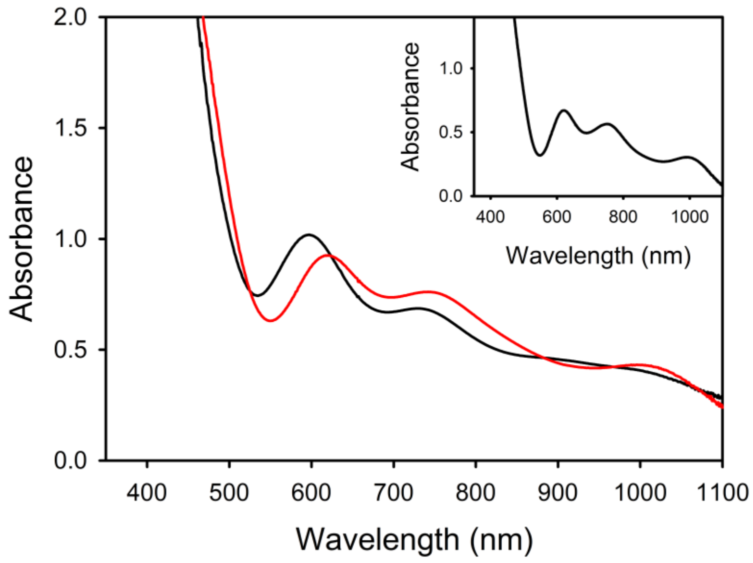

Figure S25. UV−vis−NIR spectra of [Na(15c5)][**2**] in DMSO before (black) and after addition of 1000 equiv. of propionitrile (red). Inset: the spectrum of [Na(15c5)][**2**] after reaction with 1 equiv. of [2,6−LutH][BAr^F_4].

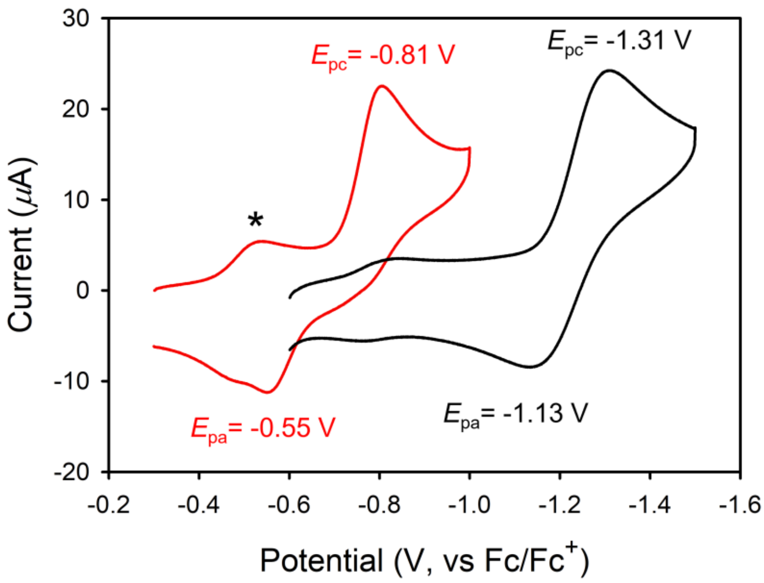

Figure S26. Cyclic voltammograms of [Na(15c5)][**2**] (black) and [Na(15c5)][**2**] with 1 equiv. of [2,6–LutH][BAr^F_4] (red) in DMSO (1.0 mM). Conditions: 0.1 M NBu_4PF_6, Pt, and Ag/$AgNO_3$ as the supporting electrolyte, working electrode, and reference electrode, respectively. Scan rate: 0.1 V/s. *: Signal corresponds to the reduction of $[Ni^{III}(PS3")]_2$. [Na(15c5)][**2**], $E_{1/2}$ (ΔE) = −1.22 (0.18) V; [Na(15c5)][**2**] with 1 equiv. of [2,6–LutH][BAr^F_4], $E_{1/2}$ (ΔE) = −0.68 (0.26) V.

Note: The protonated species **2−H**$^+$ underwent dimerization in solution, likely due to the lability of the coordinated H_2O ligand, resulting in the formation of a Ni^{III}/Ni^{III} dimer in less than 30% yield. This species was also detected in ^1H NMR data shown in **Figure S24**.

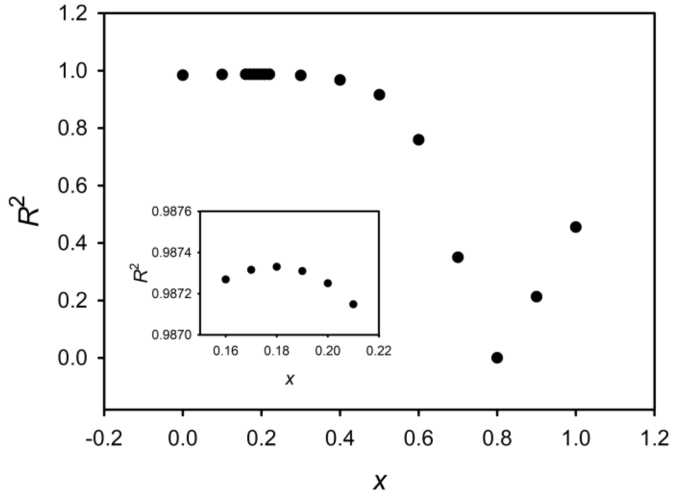

x	R^2
1	0.45549
0.9	0.21280
0.8	0.00004
0.7	0.34983
0.6	0.75921
0.5	0.91600
0.4	0.96723
0.3	0.98342
0.22	0.98700
0.21	0.98715
0.2	0.98725
0.19	0.98731
0.18	0.98733
0.17	0.98731
0.16	0.98727
0.1	0.98646
0	0.98394

Figure S27. Plot of R^2 versus x, with an expanded view of the range x= 0.15~0.22.

Reference

1. Block, E.; Ofori-Okai, G.; Zubieta, J., 2-Phosphino-and 2-Phosphinylbenzenethiols: New Ligand Types. *J. Am. Chem. Soc.* **1989,** *111* (6), 2327-2329.

2. Lee, C.-M.; Chiou, T.-W.; Chen, H.-H.; Chiang, C.-Y.; Kuo, T.-S.; Liaw, W.-F., Mononuclear Ni(II)-Thiolate Complexes with Pendant Thiol and Dinuclear Ni(III/II)-Thiolate Complexes with Ni···Ni Interaction Regulated by the Oxidation Levels of Nickels and the Coordinated Ligands. *Inorg. Chem.* **2007,** *46* (21), 8913-8923.

3. Sheldrick, G. M., SADABS. *University of Göttingen, Germany* **1996**.

4. Sheldrick, G., SHELXTL, Version 5; Siemens Analytical Xray Systems. *Inc.: Madison, WI* **1994**.

5. Gordon, J. B.; Albert, T.; Yadav, S.; Thomas, J.; Siegler, M. A.; Moënne-Loccoz, P.; Goldberg, D. P., Oxygen versus Sulfur Coordination in Cobalt Superoxo Complexes: Spectroscopic Properties, O_2 Binding, and H-Atom Abstraction Reactivity. *Inorg. Chem.* **2023,** *62* (1), 392-400.

6. Warren, J. J.; Tronic, T. A.; Mayer, J. M., Thermochemistry of Proton-Coupled Electron Transfer Reagents and its Implications. *Chem. Rev.* **2010,** *110* (12), 6961-7001.

7. Zhang, X.; Bordwell, F. G., Acidities and homolytic bond dissociation energies of the acidic carbon-hydrogen bonds in radical cations. *J. Org. Chem.* **1992,** *57* (15), 4163-4168.

8. Bordwell, F. G.; Cheng, J. P.; Bausch, M. J., Acidities of Radical Cations Derived from Cyclopentadienes and 3-Aryl-1,1,5,5-tetraphenyl-1,4-pentadienes. *J. Am. Chem. Soc.* **1988,** *110* (9), 2872-2877.

9. Agarwal, R. G.; Coste, S. C.; Groff, B. D.; Heuer, A. M.; Noh, H.; Parada, G. A.; Wise, C. F.; Nichols, E. M.; Warren, J. J.; Mayer, J. M., Free Energies of Proton-Coupled Electron Transfer Reagents and Their Applications. *Chem. Rev.* **2022,** *122* (1), 1-49.

10. Bím, D.; Maldonado-Domínguez, M.; Rulíšek, L.; Srnec, M., Beyond the classical thermodynamic contributions to hydrogen atom abstraction reactivity. *Proc. Natl. Acad. Sci. U.S.A.* **2018,** *115* (44), E10287-E10294.

11. Xue, X.-S.; Ji, P.; Zhou, B.; Cheng, J.-P., The Essential Role of Bond Energetics in C–H Activation/Functionalization. *Chem. Rev.* **2017,** *117* (13), 8622-8648.

12. Barman, S. K.; Yang, M.-Y.; Parsell, T. H.; Green, M. T.; Borovik, A. S., Semiempirical method for examining asynchronicity in metal–oxido-mediated C–H bond activation. *Proc. Natl. Acad. Sci. U.S.A.* **2021,** *118* (36), e2108648118.
